# Pain - related methylation driver genes affect the prognosis of pancreatic cancer patients by altering immune function and perineural infiltration

**DOI:** 10.3389/fgene.2025.1600883

**Published:** 2025-10-08

**Authors:** Chencheng Zhang, Weiwei Xu, Xudong Chen, Xingdan Wang, Xiaopeng Cui, Wenjing Zhao

**Affiliations:** ^1^ Cancer Research Center Nantong, Nantong Tumor Hospital & Tumor Hospital Affiliated to Nantong University, Nantong, China; ^2^ Department of Oncology, Nantong Tumor Hospital & Tumor Hospital Affiliated to Nantong University, Nantong, China; ^3^ Department of Pathology, Nantong Tumor Hospital & Tumor Hospital Affiliated to Nantong University, Nantong, China; ^4^ Department of Radiotherapy, Nantong Tumor Hospital & Tumor Hospital Affiliated to Nantong University, Nantong, China; ^5^ Department of General Surgery, Affiliated Hospital of Nantong University, Nantong, China

**Keywords:** pancreatic carcinoma, DNA methylation, perineural invasion (PNI), neuropathic pain, immune microenvironment, prognostic signature

## Abstract

**Background:**

The malignant progression of pancreatic cancer (PC) is frequently accompanied by intractable pain mediated through perineural invasion (PNI), yet the underlying epigenetic regulatory mechanisms remain elusive.This study aims to elucidate the role of DNA methylation in the pathogenesis of PC pain, including its interactive effects with the nervous and immune systems.

**Methods:**

Integrating multi-omics data from TCGA-PAAD (Pancreatic adenocarcinoma), we identified methylation driver genes (MDGs) using the MethylMix algorithm. By intersecting MDGs with pain-related gene sets and conducting multi-step regression modeling, we established a five-gene prognostic signature (PSMB8/COL17A1/BICC1/CTRC/TRIP13). Next, in order to elucidate the underlying mechanisms, we conducted differential expression analysis, protein-protein interaction network analysis, functional enrichment analysis, and single-cell sequencing. Additionally, we quantified immune infiltration using CIBERSORT and TIMER.

**Results:**

Pain-related MDGs are enriched in immune regulation, extracellular matrix reorganization, and cation channel activity, constituting the “immune-neural axis” of epigenetic regulation. The prognostic five-gene signature significantly stratifies patient survival risk (HR = 3.83, p = 1.4e−8), with its methylation levels positively correlated with CD4^+^ T cell infiltration and negatively correlated with dendritic cells. Model-derived differentially expressed genes exhibited dual immune-neural tropism at single-cell resolution, prominently enriched in presynaptic signaling and synaptic vesicle cycling. Mechanistically, MDGs orchestrate pain progression through PNI-associated neural remodeling and K+ channel-mediated neuronal hypersensitization.

**Conclusion:**

This study establishes a visceral pain model centered on pancreatic parenchymal nociception rather than secondary neural effects, and for the first time proposes an interconnected regulatory network linking epigenetic modifications, immune reprogramming, and neural plasticity, revealing dual pain pathogenesis mechanisms: (1) immune microenvironment reshaping that potentiates neuroinflammation, and (2) direct ion channel regulation enhancing neuronal excitability. These findings provide a mechanistic foundation for developing methylation-based prognostic biomarkers and multimodal analgesic therapeutic strategies targeting the immuno-neural nexus.

## 1 Introduction

The global incidence of PC continues to rise, characterized by its high malignancy and early-stage concealment. Over 80% of patients are diagnosed at an advanced stage, losing the opportunity for curative surgery. The high postoperative metastasis rate and a 5-year survival rate of less than 10% place PC as the fourth leading cause of cancer-related deaths worldwide (Kalra and Meltzer, 2025). Pain, as a primary symptom, affects approximately 90% of patients, significantly reducing quality of life and potentially triggering anxiety, depression, and even suicidal tendencies (Tarasiuk et al., 2023; Shrestha et al., 2024). In 2018, the WHO for the first time classified “chronic cancer pain” as a distinct disease category (Bennett et al., 2019), with effective pain management shown to prolong survival (Ozcan et al., 2019). However, the mechanisms underlying pain remain unclear, and clinical management relies heavily on empirical approaches, highlighting the urgent need for molecular mechanism research (Cai et al., 2021; Damm et al., 2020).

The study found that the main cause of PC pain is neuropathic pain triggered by tumor cells invading nerves, which is considered a result of PNI (Zhu et al., 2024). PNI defined as cancer cells invading along nerves or the interstitial spaces of the neural sheath, perineurium, and endoneurium, is a special way for cancer to spread to distant sites (Zhu et al., 2024). The incidence of PNI in PC is nearly 100%, which is negatively related to the survival rate, and is a risk factor for R1 resection and recurrence (Selvaggi et al., 2022; Ozaki et al., 1999). The sensory nerve endings distributed within internal organs, such as the pancreas, and blood vessel walls are capable of perceiving stimuli including osmotic pressure, temperature, and pathological injuries (Drewes et al., 2020). Pain signals are transmitted *via* nerve fibers to the cell bodies of cranial/spinal ganglia (Jiang et al., 2025), and ultimately ascend to the central nervous system through afferent nerves. This conduction pathway confirms that visceral pain signals originate from pathological changes within the parenchyma of the organs (Ten Barge et al., 2025). However, previous studies on PNI-related pain models in PC have primarily focused on the sciatic nerve and dorsal root ganglia affected by tumor cells, concentrating on the pain signal transmission process while ignoring the initial site of pain generation in the viscera (Miura et al., 2018).

Recent studies have found that Schwann cells (key components of peripheral nerves) are significantly enriched in pancreatic tumor tissues (Xue et al., 2023). By interacting with tumor/immune/stromal cells, Schwann cells activate tumor-neural system dialogue, promoting metastasis and driving the process of carcinogenesis (Xu W. et al., 2024). On the one hand, tumor cells obtain more growth signals, remodel metabolism, and evade immune surveillance to promote survival by inducing their own innervation (Hirth et al., 2020). When PC cells invade nerves, they disrupt the normal structure of nerve fibers, leading to abnormal nerve signal transmission (Yang et al., 2024). Abnormal nerve impulses are transmitted along damaged nerve fibers, triggering pain sensations. During PNI, PC secretes nerve growth factors such as NGF, which may increase the sensitivity of nerve endings, known as hyperalgesia (Xu J. et al., 2024; Liu et al., 2024). PNI in PC may also lead to changes in neural plasticity, including nerve hyperplasia and hypertrophy, which further exacerbate abnormal nerve signal transmission and hyperalgesia (Xu et al., 2023). On the other hand, the sympathetic nerves, parasympathetic nerves, and sensory nerves within the pancreas voluntarily adapt to the progressive malignant process and establish bidirectional communication with the tumor to support its growth (Feng et al., 2021). Besides direct effects, nerves regulate the progression of PC by actively regulating the functions of stroma and immune components, and the involvement of tumor microenvironment (TME) complicates the bidirectional interaction (Chen et al., 2020). Taking the immune system as an example, immune cell activation and mediator release induce/maintain cancer pain (Bethea and Fischer, 2021), and pain-related immune disorders (mainly inflammation) and immunosuppression delay pain resolution (Zhao et al., 2023), ultimately impairing patients’ immune function and prognosis. Combined with previous studies, it was found that the immune system and nervous system participate in regulating the occurrence and development of pain. However, little is known about how the two systems are related to the initial signal of pain generation.

In the study of pain-related mechanisms, epigenetic modifications involving DNA methylation have also garnered significant attention. In neuropathic pain models, DNMT-mediated hyper-methylation of the Oprm1/Kcna2 gene promoters leads to gene silencing, which triggers increased neuronal excitability and hyperalgesia (Sun et al., 2017; Sun et al., 2019). In rat dorsal root ganglion (DRG), DNMT1-driven downregulation of Cnr1 gene methylation can weaken its inhibitory effect on TRPV1, exacerbating visceral pain (Hong et al., 2015). In CFA-induced chronic inflammatory pain, DNMT3b inhibition-induced de-methylation of the NGF promoter upregulates NGF expression and maintains the pain state by promoting C/EBPα binding (Yuan et al., 2020). Although oral cancer studies suggest that antitumor gene de-methylation and neurotrophin hyper-methylation are involved in the PNI process (Hurnik et al., 2022), the intrinsic association between DNA methylation and PNI in PC pain remains unknown.

**FIGURE 1:**
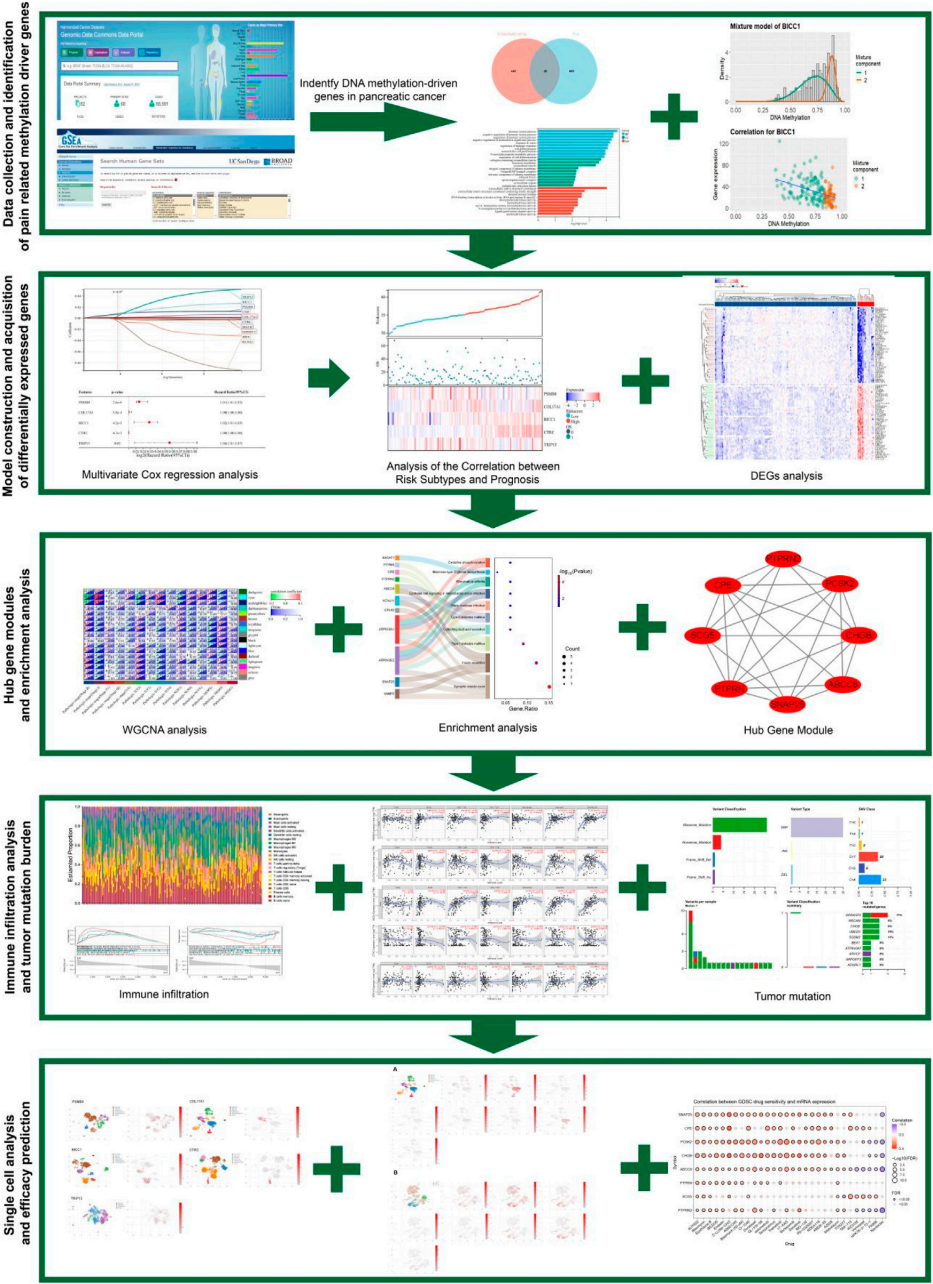
The flowchart of this article.

This study integrated TCGA PC methylation data with the MSigDB pain gene set, and identified 26 pain-related MDGs using the MethylMix algorithm to construct a pain risk scoring model. After stratifying patients based on this model and combining it with single-cell analysis, it was revealed that tumor-derived pain signals drive neuroplastic changes and immune microenvironment reprogramming through PNI, leading to a molecular axis of poor prognosis (Figure 1). This study innovatively focuses on the primary visceral pain site (distinct from secondary neural effects) and systematically analyzes the complete mechanism of pain signaling from its initial generation to transmission. It provides a theoretical foundation for the early diagnosis of PC pain and targeted multi-modal analgesic strategies for the “immune-neural hub”.

## 2 Materials and methods

### 2.1 Data collection

We downloaded RNA sequencing data, metabolomics data, somatic mutation data, and The Cancer Genome Atlas (TCGA; https://portal.gdc.cancer.gov/repository) (Zhang and Wang, 2015) from 178 PC tissues and four normal pancreatic tissues. The pain-related gene set HP_PAIN (M38128) was obtained by searching the keyword “pain” from the Molecular Signatures Database (MSigDB), which contains 835 genes related to pain (MSIGDB_URL:https://www.gsea-msigdb.org/gsea/msigdb/human/geneset/HP_PAIN). The mRNA expression profiles and clinical information of the GSE183795 dataset were downloaded from the GEO database as a validation cohort. This dataset includes microarray gene expression profiles of 139 pancreatic tumors, 102 adjacent non-tumor tissues, and three normal pancreases from donors with pancreatic ductal adenocarcinoma patients.

### 2.2 Identify pain - related MDGs in PC and conduct functional enrichment analysis

By using the beta hybrid model of the MethylMix package, we can identify sample subpopulations of PC with different DNA methylation compared with normal tissues, so as to identify differential and functional DNA methylation. Functional DNA methylation refers to 471 MDGs of PC based on significant negative correlation of matched gene expression data. By intersecting with pain-related genes in the MsigDB database, 26 pain-related MDGs were ultimately obtained.

Conduct Gene Ontology (GO) analysis and Kyoto Encyclopedia of Genes and Genomes (KEGG) analysis on 26 genes, and analyze gene characteristics from aspects of molecular function, biological process and cellular component as well as the interactions and regulations of their pathways in biological systems.

### 2.3 Construction and validation of risk scoring model

In this study, using the TCGA cohort, we employed the R package glmnet to integrate survival time, survival status, and gene expression data, and utilized the lasso-cox method for regression analysis. Additionally, we set up 10-fold cross-validation to obtain the optimal model. We set the λ value to be 0.0675911321389309, and finally obtained a model formula constructed by 10 genes as follows: RiskScore = 2.94730303909491e-05*CTRC+0.0038277720604039*TRIP13+0.00767681073907178*PSMB8-0.00156609272886264*IRF4+5.34857478531571e-05*GNE+0.00651372422293902*BICC1+0.00119961881628371*COL17A1-0.00169964562145739*FERMT3-0.00549988980019879*KCNJ2-0.000188824126427788*MAFB.

After multivariate survival analysis, five pain-related MDGs were found to be significantly associated with overall survival. These five genes are CTRC, TRIP13, PSMB8, BICC1, and COL17A1. We used the R package maxstat (Maximally selected rank statistics with several p-value approximations version: 0.7-25) to calculate the optimal cutoff value of RiskScore. We set the minimum number of samples in a group to be greater than 25% and the maximum number of samples in a group to be less than 75%. Finally, the optimal cutoff value was obtained as 1.81837560981286. Based on this, patients were divided into high-risk and low-risk groups. Then, we further used the survfit function in the R package survival to analyze the prognostic differences between the two groups, and the logrank test method was used to evaluate the significance of prognostic differences between different groups of samples. Eventually, we observed significant prognostic differences (p = 1.4e−8). Analyzed the relationship between risk scoring and pathological grading and staging, and observed the survival curves of the five main pain-related MGDs that make up the risk scoring model We selected the mRNA expression profiles and clinical information of the GSE183795 dataset downloaded from the GEO database as the validation cohort to observe the survival curves and prognostic analysis of high-risk and low-risk subgroups, thus verifying the risk score model.

### 2.4 Analysis of key pain-related MDGs

MethylMix is an algorithm for identifying highly methylated and hypo-methylated genes associated with diseases. MethylMix identifies methylation states based on the β-mixture model and compares them with normal DNA methylation states. MethylMix uses a new statistical quantity, namely, the difference methylation value or DM value, which is defined as the difference between the methylation state and the normal methylation state. Finally, the matched gene expression data are used to identify functional methylation states other than differences by focusing on the methylation changes that affect gene expression. This study, based on the TCGA PC gene expression matrix and DNA methylation, analyzed 471 PC MDGs by using the MethylMix package. The methylation states of five key pain-related MDGs and their correlations with mRNA expression levels were analyzed, and the gene expression differences in tumors and normal tissues were also analyzed.

### 2.5 Differential gene selection and weighted gene Co-expression network analysis (WGCNA)

Based on the risk score model, TCGA PC patients were divided into high-risk and low-risk subtypes. Limma is a differential expression screening method based on the generalized linear model. Here, we used the R package limma (version 3.40.6) for differential analysis to obtain differentially expressed genes between the high-risk and low-risk subtypes. A total of 5,962 differentially expressed genes were identified with a fold change of 1.5 and p < 0.05, including 3,886 upregulated genes and 2,076 downregulated genes. After analyzing their chromosomal locations, all differentially expressed genes underwent WGCNA to identify differentially expressed genes in the most relevant modules. Combined with survival time and survival status, the most relevant module cyan was determined, which encompassed 89 genes. Enrichment analysis of GO and KEGG was performed on the genes in this module.

### 2.6 Construction of protein-protein interaction (PPI) networks and identification of hub genes

To study the differentially expressed genes associated with pain risk subtypes in PC methylated driver genes, we input the selected cyan module genes into the String database to construct a PPI network, with a cutoff value set at 0.400, and visualized it using Cytoscape software. Subsequently, we utilized the Molecular Complex Detection (MCODE) tool in Cytoscape to analyze the gene interaction information. By applying criteria of degree cutoff = 2, node score cutoff = 0.2, and K-Core = 2, we identified hub modules within the PPI network, comprising eight genes, and conducted a visualization analysis of their gene functions.

### 2.7 Analysis of tumor mutation burden

Tumor Mutational Burden (TMB) as an emerging biomarker, can assist in predicting patients’ response to immunotherapy. In this study, we analyzed the mutational landscape of the top 20 most frequently mutated genes in the pain-related risk subtypes of PC patients, demonstrating the specific mutation conditions of all samples. With the help of “maftools” package in R software, we calculated the tumor mutation conditions of PC patients. The “survival” package was used to determine the optimal cut-off value, and the samples were divided into high TMB group and low TMB group based on TMB differential analysis. Subsequent survival analysis was conducted regarding the TMB.

### 2.8 Immune cell infiltration and gene set enrichment analysis

IOBR (Zeng et al., 2021) is a computing tool for immune tumor biology research. Here, based on the expression profile of TCGA PC patients, the R software package IOBR selected xCell method to calculate 64 immune cell infiltration scores and immune, matrix and microenvironment scores for each sample. The CIBERSORT method calculated 22 immune cell infiltration scores for each sample and analyzed the correlation between immune cell infiltration scores and 10 hub genes. For Gene Set Variation Analysis (GSVA), we used the R software package to calculate the enrichment score of each sample in the gene set from GSVA (version 1.40.1). We predefined the gene rank. Specifically, we first used gene expression to reach the spectrum, using the Hanzelmann et al. method, and downloaded the hallmark subset from the Molecular Signatures Database (http://www.gsea-msigdb.org/gsea/downloads.jsp) to evaluate relevant pathways and molecular mechanisms, Set the minimum gene set to 5 and the maximum gene set to 5,000, and calculate the enrichment scores of each sample in each gene set. Finally, the enrichment score matrix was obtained.

### 2.9 Analysis of the correlation between gene methylation, copy number variation and immune infiltration

GSCALite is a web-based platform for gene set cancer analysis. GSCALite integrates cancer genomics data of 33 cancer types from TCGA, drug response data from GDSC and CTRP, and normal tissue data from GTEx for an integrated data analysis workflow in gene set analysis. In this study, the key genes under investigation are input into the GSCALite platform to analyze their interrelationships with immune infiltration. TIMER is a comprehensive database whose primary function is to systematically analyze six types of tumor-infiltrating immune cells (B cells, CD4^+^ T cells, CD8+T cells, neutrophils, macrophages, and dendritic cells) in different cancer types through the TIMER algorithm. At the same time, it analyzes the relationship between gene expression and tumor purity. Genes that are highly expressed in the tumor microenvironment are negatively correlated with tumor purity, while genes that are highly expressed in tumor cells are positively correlated with tumor purity.

### 2.10 Single-cell sequencing analysis

CDCP is a comprehensive platform for single-cell data integration, sharing and analysis. Users can obtain detailed information about samples in the datasets included in the CDCP single-cell data platform online, and are allowed to use tSNE (t-Distributed Stochastic Neighbor Embedding) cell dimensionality reduction maps and clustering analysis maps of different cell types to visualize each single-cell dataset. In this study, the CDCP online platform was utilized to analyze the gene expression of key genes in single cells in the pancreas or nerves and visualize them for comparison.

## 3 Results

### 3.1 Construction and validation of risk scoring model for pain-related MDGs in PC

**FIGURE 2:**
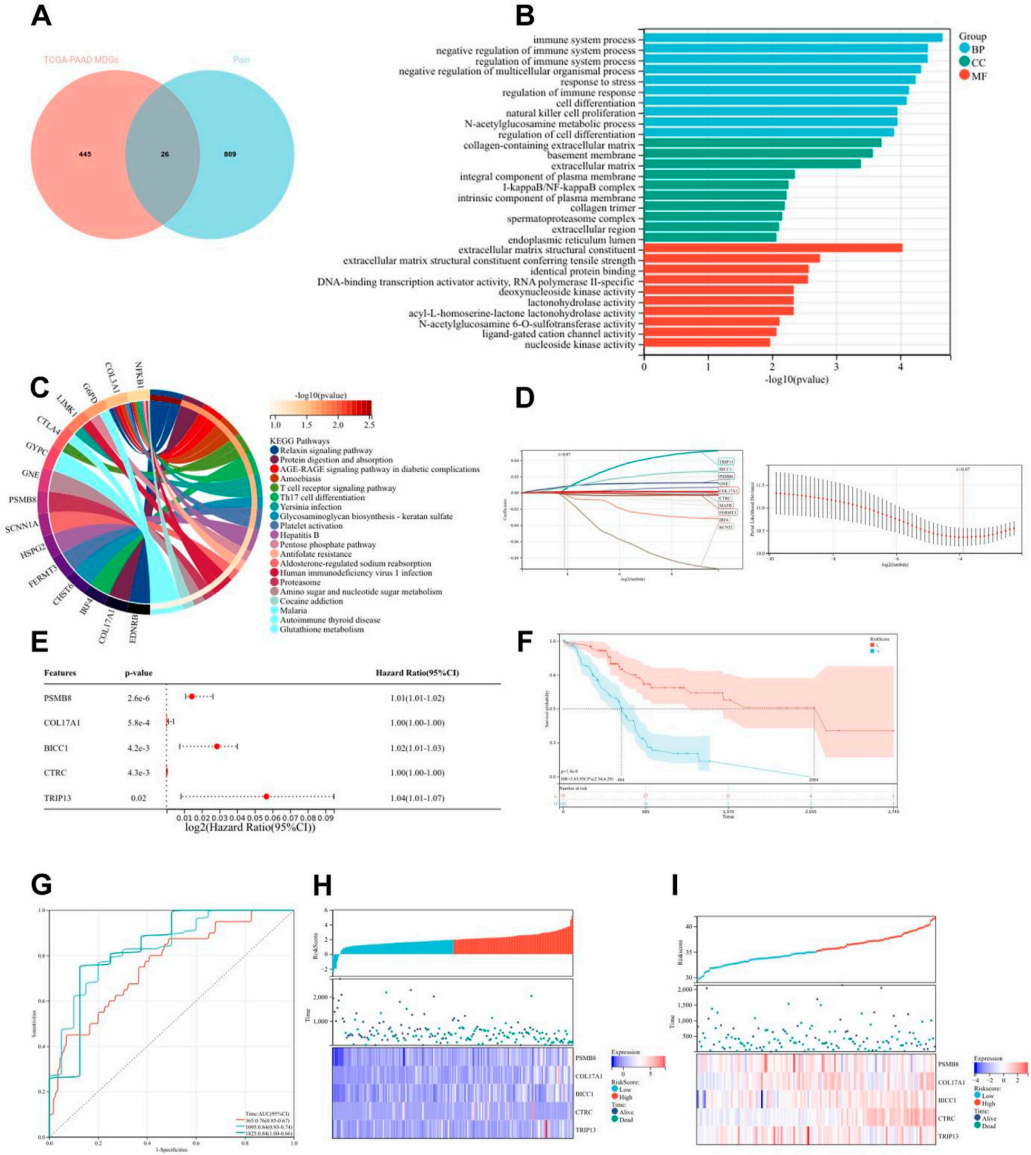
Construction and validation of risk scoring model for pain-related MDGs in PC. **(A)** 26 pain-related MDGs of PC were obtained by intersection of PC MDGs and pain gene collection in MSigDB. **(B)** GO enrichment analysis of 26 pain-related MDGs in PC. **(C)** KEGG enrichment analysis of 26 pain-related MDGs in PC. **(D)** Using the R software package glmnet, integrate survival time, survival status, and gene expression data, and perform regression analysis using lasso cox method to obtain 10 genes, the λ value is 0.0675911321389309 for. **(E)** Forest plot of 5 pain-related MDGs with significant differences obtained from multivariate Cox regression analysis. **(F)** KM curve of pain risk scoring model. **(G)** ROC curve of pain risk scoring model. **(H)** Prognostic heatmap analysis of pain risk scoring model. **(I)** Prognostic heatmap analysis of pain risk scoring model in GSE183795 dataset.

Emerging evidence has established epigenetic regulation, particularly DNA methylation, as a central modulator of chronic pain pathophysiology (Jiang et al., 2022). To systematically investigate methylation-driven mechanisms underlying PC-associated pain, we performed multi-dimensional bioinformatics analysis of PC samples from TCGA (n = 178). High-resolution methylation arrays and RNA-seq data were integrated through the MethylMix algorithm, revealing 471 aberrantly methylated driver genes (FDR < 0.05) with 333 hypermethylated and 138 hypomethylated candidates (Supplementary Table 1). Cross-referencing these epigenetic drivers with a curated database of 835 nociception-associated genes (PainGenesDB v4.0) identified 26 high-confidence pain-related MDGs (15 hypermethylated, 11 hypomethylated; Figure 2A; Supplementary Tables 2, 3). GO enrichment analysis revealed that these 26 genes are enriched in extracellular matrix structural constituents, DNA-binding transcription activator activity, ligand-gated cation channel activity, and other molecular functions. In biological processes, they are enriched in the regulation of immune system processes, immune function modulation, and cell differentiation regulation. In terms of cellular components, they are enriched in the extracellular matrix and integral components of the plasma membrane (Figure 2B). KEGG enrichment analysis showed that these genes are associated with signaling pathways such as T cell receptor signaling pathway and Th17 cell differentiation (Figure 2C). Thus, it can be seen that PC pain-related MDGs may play a role in immune response and cell differentiation and proliferation, suggesting an intrinsic link between methylation and pain in PC.

The lasso-cox regression analysis was conducted on the screened 26 pain-related MDGs, followed by a 10-fold cross-validation to obtain the optimal risk scoring model (Figures 2D,E). The λ value was determined to be 0.0675911321389309. The formula for the model constructed using the final 10 selected genes is as follows:

RiskScore = 2.94730303909491e-05*CTRC+0.0038277720604039*TRIP13+0.00767681073907178*PSMB8-0.00156609272886264*IRF4+5.34857478531571e-05*GNE+0.00651372422293902*BICC1+0.00119961881628371*COL17A1-0.00169964562145739*FERMT3-0.00549988980019879*KCNJ2-0.000188824126427788*MAFB.

After multivariate survival analysis, five pain-related MDGs were identified as significantly correlated with overall survival (Figure 2F). These five genes are: PSMB8, COL17A1, BICC1, CTRC, and TRIP13. The hazard ratio (HR) is 3.83. Using the R package “maxstat”, the optimal cutoff value for RiskScore was calculated to be 1.81837560981286. Based on this value, patients were divided into high and low groups, showing significant prognostic differences (p = 1.4e−8) (Figure 2G). The ROC curve revealed that the AUCs for patient survival at 1, 3, and 5 years were 0.76, 0.84, and 0.84, respectively (Figure 2H). The prognostic heatmap analyzed the relationship between different risk scores and patient follow-up time, events, and changes in gene expression. It was observed that as the risk score increased, the survival rate of patients decreased (Figure 2I). The risk scoring model was validated using the mRNA expression profiles and clinical information from the GSE183795 dataset as a validation cohort (Figure 2J).The HR of GSE183795 is 1.95 (p = 2.3e−3), and the ROC curve reveals that the AUCs for patient survival at 1, 3, and 5 years were 0.55, 0.64 and 0.67, respectively, which is significant (Supplementary Figure 1).

### 3.2 Risk model is significantly related to pathological staging of PC

**FIGURE 3:**
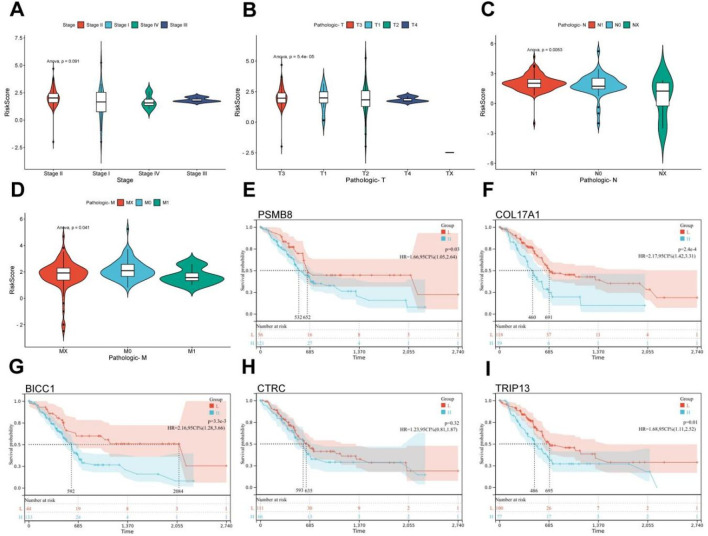
Clinical relevance and gene survival curve of risk model. **(A)** Correlation between TCGA PC patient grade and risk score. **(B)** Correlation between T stage and risk score in TCGA patients with PC. **(C)** Correlation between N stage and risk score in TCGA PC patients. **(D)** Correlation between M-stage and risk score of TCGA PC patients. **(E)** Kaplan Meier curve of PSMB8. **(F)** Kaplan Meier curve of COL17A1. **(G)** Kaplan Meier curve of BICC1. **(H)** Kaplan Meier curve of CTRC. **(I)** Kaplan Meier curve of TRIP13 (ANOVA, P ≤ 0.05).

Based on the tumor staging and TNM staging information of PC patients from TCGA, combined with the risk score derived from pain-related MDGs, the correlation between the risk score and the clinical information of PC was evaluated. In the tumor staging of PC, stage Ⅰ tumors generally do not have regional lymph node metastasis or distant metastasis, while stage Ⅱ and Ⅲ tumors have different degrees of regional lymph node metastasis, and stage Ⅳ tumors are generally accompanied by distant metastasis. As can be seen from the results in Figure 3A, the high-risk score is most correlated with PC stage Ⅱ. Stage Ⅱ tumors have 1–3 regional lymph node metastases. It is speculated that pain-related MDGs play an important role in the early metastasis process of tumors. Observing the results of the correlation between the TNM staging and the risk score of PC patients, it can be seen that pain is correlated with the size of the primary tumor (T staging) and the situation of regional lymph node metastasis to different degrees. No significant association with distant metastasis (Figures 2B–D). It can be seen that the risk score model constructed in this study is more suitable for the stage where PC begins to spread but has not yet had distant metastasis. A high-risk score indicates that the tumor has a tendency to metastasize. The Cox multivariate analysis obtained five key pain-related MDGs. The Kaplan-Meier curves of these five genes in TCGA PC patients were drawn respectively, and it can be seen that PSMB8, COL17A1, BICC1 and TRIP13 are significantly correlated with the prognosis (Figures 2E–I). The metastatic propensity signature (high COL17A1/PSMB8, low CTRC) suggests: PNI facilitation via collagen remodeling (COL17A1), immune-evasion priming through proteasomal antigen processing (PSMB8),protective trypsin depletion in tumor microenvironment (CTRC).

### 3.3 Characterization of key pain-related DNA MDGs

**FIGURE 4:**
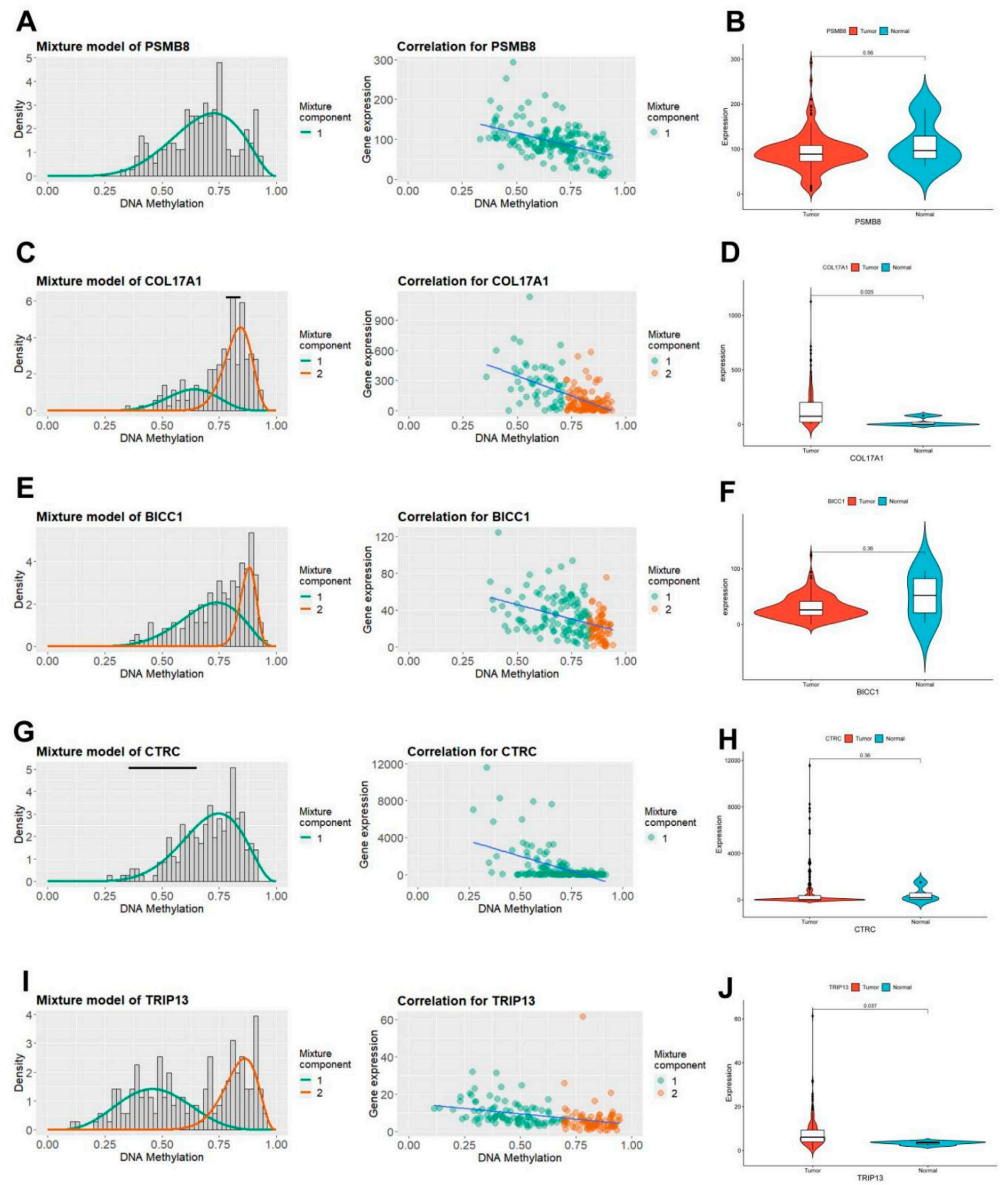
Characteristics of five pain-related epigenetic driver genes. **(A)** The methylation level of the PSMB8 gene (left) and the correlation diagram between its mRNA expression and DNA methylation level (right). **(B)** Differential mRNA expression of the PSMB8 gene in TCGA PC tissue and normal pancreatic tissue. **(C)** The methylation level of the COL17A1 gene (left) and the correlation diagram between its mRNA expression and DNA methylation level (right). **(D)** Differential mRNA expression of the COL17A1 gene in TCGA PC tissue and normal pancreatic tissue. **(E)** The methylation level of the BICC1 gene (left) and the correlation diagram between its mRNA expression and DNA methylation level (right). **(F)** Differential mRNA expression of the BICC1 gene in TCGA PC tissue and normal pancreatic tissue. **(G)** The methylation level of the CTRC gene (left) and the correlation diagram between its mRNA expression and DNA methylation level (right). **(H)** Differential mRNA expression of the CTRC gene in TCGA PC tissue and normal pancreatic tissue. **(I)** The methylation level of the TRIP13 gene (left) and the correlation diagram between its mRNA expression and DNA methylation level (right). **(J)** Differential mRNA expression of the TRIP13 gene in TCGA PC tissue and normal pancreatic tissue.

The MethyMix algorithm can identify genes’ low/high methylation states through the beta mixture model and find DNA MDGs in diseases by analyzing the correlation of the expression levels of corresponding genes. The expression patterns (left) and negative correlation maps (right) of key pain-related MDGs in different samples are shown in Figure 4 (PSMB8: Figure 4A, COL17A1: Figure 4C, BICC1: Figure 4E, CTRC: Figure 4G, TRIP13: Figure 4I). Combined with the differential methylation values of each driver gene (it’s the average difference in methylation between tumor samples and normal samples, Supplementary Table 4), it can be seen that PSMB8 and CTRC are not methylated in normal samples of PC in the TCGA database. Observing the mRNA expression levels of key pain-related MDGs in pancreatic tumor and normal samples, significant differences can be seen in the mRNA expression levels of PSMB8 and TRIP13 (PSMB8: Figure 4B, COL17A1: Figure 4D, BICC1: Figure 4F, CTRC: Figure 4H, TRIP13: Figure 4J).

### 3.4 Differential gene identification and WGCNA of risk scoring model

**FIGURE 5:**
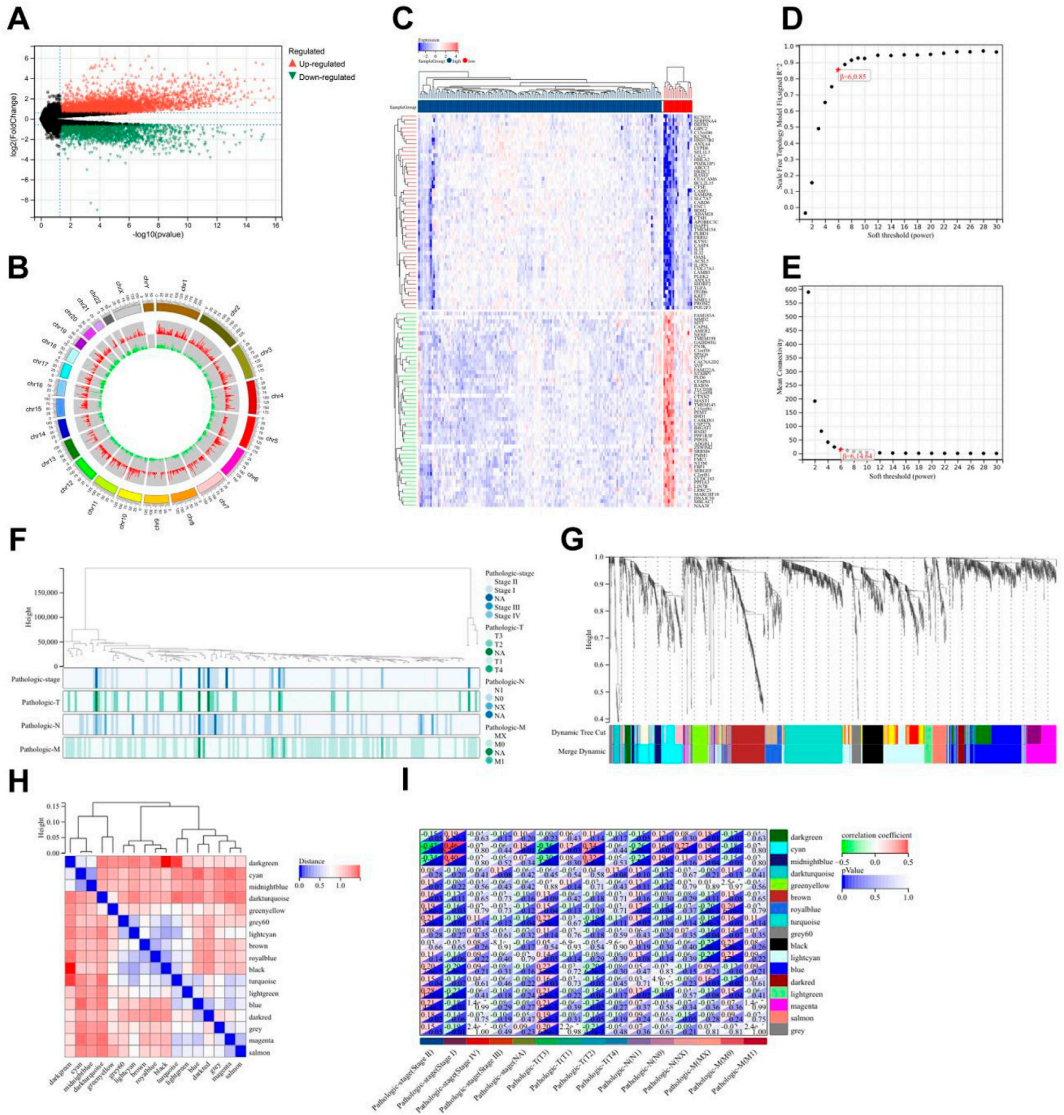
Differential gene analysis of different risk subtypes. **(A)** Volcano plot of differentially expressed genes related to pain, using |logFC| > 1.5 and p-value < 0.05 as screening criteria. **(B)** Chromosome locations of 5,962 differentially expressed genes. **(C)** Heatmap of the top 50 differentially expressed genes. **(D)** Scale-free topology model fitting of 5,962 differentially expressed genes analyzed by WGCNA. **(E)** Average connectivity of differentially expressed genes analyzed by WGCNA. **(F)** Sample clustering of differentially expressed genes analyzed by WGCNA. **(G)** Gene clustering of differentially expressed genes analyzed by WGCNA. **(H)** Module eigengene clustering of differentially expressed genes analyzed by WGCNA. **(I)** Heatmap of correlation between WGCNA analysis modules of differentially expressed genes and phenotypes.

In the TCGA cohort, limma analysis identified a total of 3,886 upregulated and 2,076 downregulated differentially expressed genes (DEGs) (|logFC| > 1.5 and p-value < 0.05) between the two pain-related risk subtypes (Figure 5A; Supplementary Table 5). Chromosomal mapping revealed chromosome 19 enrichment (q = 0.003) of upregulated genes, while downregulated genes clustered on 7q21.3 (PAX/POU domain loci; Figure 5B). The heatmap visualization of the first 50 differentially expressed genes is shown in Figure 5C. The goodSamplesGenes method in the R package WGCNA was used to remove outlier genes and samples, and then the scale-free co-expression network was further constructed using WGCNA. Pearson correlation matrix and average linkage method were applied to all pairs of genes, and then a weighted adjacency matrix was constructed. The adjacency relationship was transformed into a topological overlap matrix (TOM), which can measure the network connectivity of a gene, defined as the sum of its adjacency relationships with all other genes, for network gene ratio, and the corresponding dissimilarity (1-TOM) was calculated (Figures 5D,E). Genes with similar expression profiles were classified into gene modules. According to the dissimilarity measure based on TOM, average linkage hierarchical clustering was carried out, and the minimum size (gene group) of the gene dendrogram was set to be 30 (Figures 5F,G). The sensitivity was set as 3. In order to further analyze the modules, the dissimilarity of characteristic genes of the modules was calculated, a cutting line was selected for the module dendrogram, and some modules were merged. In addition, modules with a distance less than 0.25 were also merged. Finally, 17 co-expression modules were obtained, among which the Gray module was considered as unable to be reasonably combined with any other model. Visualizing the correlations of the 17 co-expression gene modules resulted in Figure 5H, from which it can be seen that the darkgreen module has the highest correlation with the black module. By comprehensively analyzing the correlations of the 17 co-expression gene modules with tumor staging, it can be seen that the cyan module is most correlated with tumor stage and TNM staging. Therefore, it was selected as the key module for subsequent analysis, which contains a total of 89 genes (Figure 5I; Supplementary Table 6).

### 3.5 Enrichment analysis of key module genes and construction of protein-protein interaction networks

**FIGURE 6:**
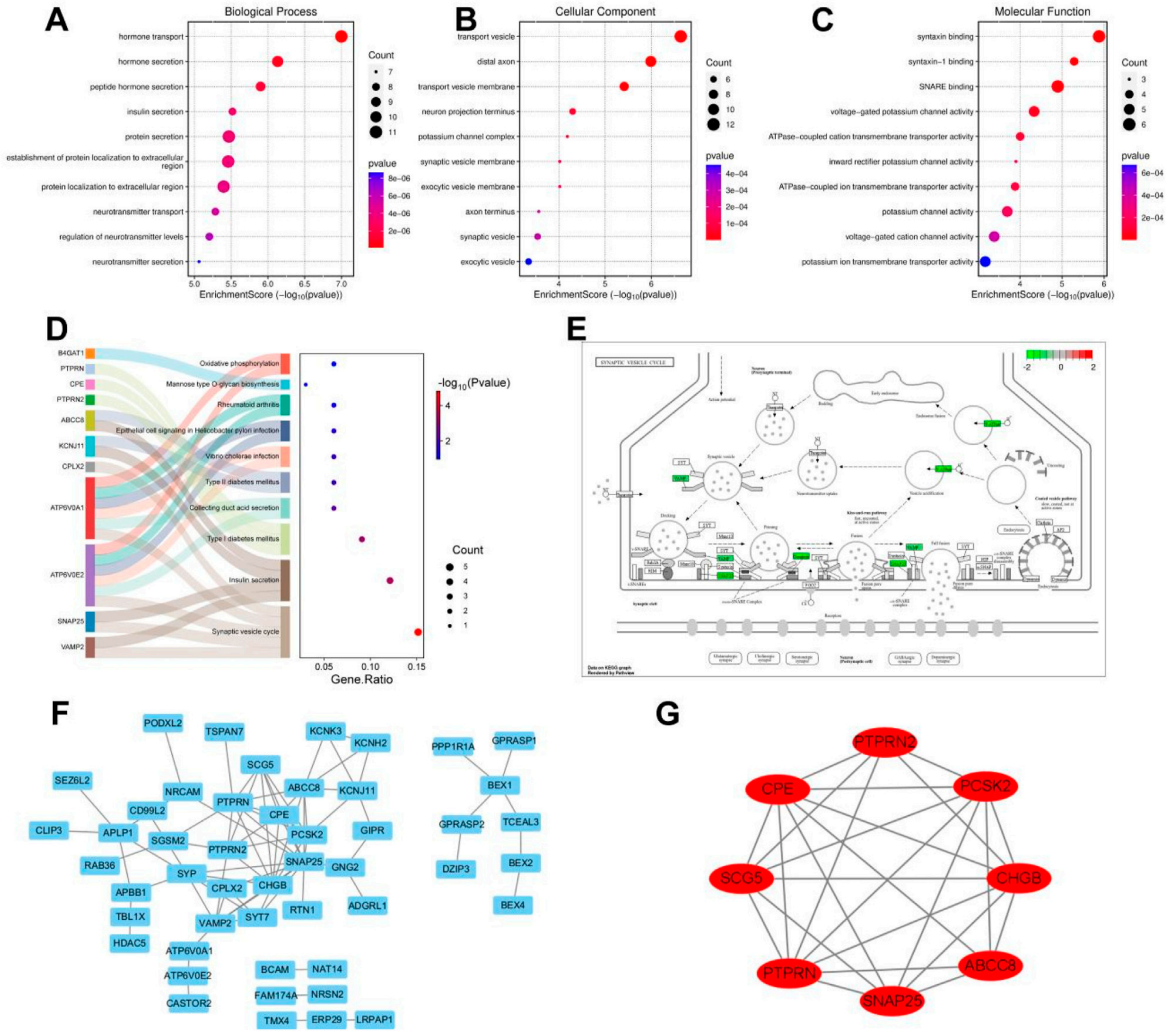
Enrichment analysis of key module genes and construction of protein-protein interaction network. **(A)** Biological processes of GO enrichment analysis in the cyan module. **(B)** Cellular components of GO enrichment analysis in the cyan module. **(C)** Molecular functions of GO enrichment analysis in the cyan module. **(D)** Top 10 pathways of KEGG enrichment analysis in the cyan module. **(E)** Genes involved in synaptic vesicle cycling in the cyan module. **(F)** Cytoscape software visualizes the protein-protein interaction network of the cyan module. **(G)** Cytoscape software visualizes key genes of the cyan module.

Subsequently, GO and KEGG enrichment analyses were performed on the crucial cyan module. The analysis results of biological processes indicated that the genes in the cyan module were concentrated on the establishment of extracellular protein localization and the secretion and transport of hormones, including insulin, neurotransmitters, and peptide hormones (Figure 6A). The analysis results of cellular components showed that the genes in the cyan module were focused on the process of signal transduction, including neuronal projection terminals, axon terminals, external vesicle membranes, synaptic vesicle membranes, transport vesicle membranes, potassium channel complexes, *etc.*, all of which are closely related to signal transduction in the nervous system (Figure 6B). The molecular function analysis results of the cyan module genes revealed a close correlation with ATPase-coupled ion transmembrane transporter activity, potassium channel activity, voltage-gated cation channel activity, and synaptotagmin binding (Figure 6C). The KEGG analysis results demonstrated that the genes in the cyan module were concentrated on oxidative phosphorylation, mannose-type O-glycan biosynthesis, and diabetes or synaptic vesicle recycling-related aspects (Figure 6D). Among them, the pathogenic mechanisms related to sugar metabolism and diabetes are closely associated with pancreatic tissues, while synaptic vesicle recycling-related functions account for the largest proportion in the KEGG analysis of the cyan module, which is consistent with the results of GO enrichment analysis. By visualizing the synaptic vesicle recycling process and marking the genes involved in the cyan module in green, it can be seen that the genes in the cyan module participate in multiple key steps of synaptic vesicle recycling in signal transduction and are crucial for signal transduction (Figure 6E). The PPI network diagram of the cyan module genes was obtained by importing them into the Cytoscape software (Figure 6F). Using the MCODE module in the Cytoscape software to contract the genes, eight key differentially expressed genes were obtained, namely, PTPRN2, CPE, SNAP25, ABCC8, PTPRN, CHGB, SCG5, and PCSK2, these genes are all downregulated in the risk scoring model (Figure 6G).

### 3.6 Gene diversity of TCGA PC and single cell analysis of key MDGs

**FIGURE 7:**
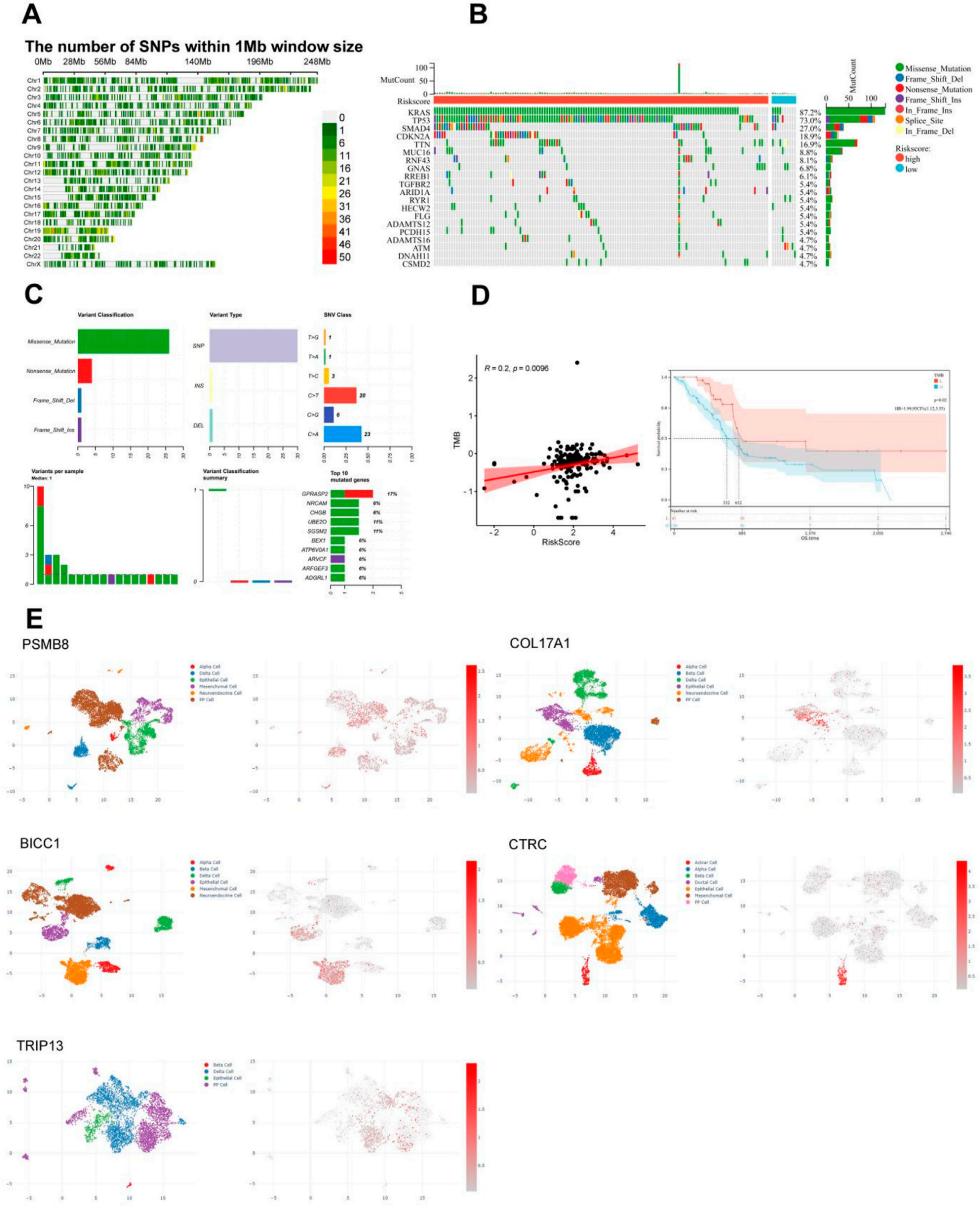
Correlation between single nucleotide polymorphism distribution, mutation status, and immunoinfiltration in TCGA PC. **(A)** Distribution of single nucleotide density in TCGA PC patients. **(B)** Waterfall diagram of somatic mutation in TCGA PC. **(C)** Summary of Gene Somatic Mutations in the cyan Module. **(D)** Correlation between tumor mutation load and risk score or survival of TCGA PC. **(E)** Results of single-cell analysis for the five pain-associated driver genes, It’s PSMB8 (GSM6567161), COL17A1 (GSM6567159), BICC1 (GSM6603326), CTRC (GSM7289740), and TRIP13 (GSM6603329).

The diversity of biological DNA sequences plays a crucial role in the occurrence and development of diseases by affecting gene expression products or gene regulatory processes, resulting in different traits in individuals. Single nucleotide polymorphism (SNP) is a major form of genetic diversity and can occur in both coding and non-coding regions of the genome. We utilized the CMplot package to visualize the distribution of SNPs in the genomes of PC patients (Figure 7A). Subsequently, we identified the top 2 genes with the highest mutation rates among TCGA PC patients (Figure 7B). It is noteworthy that mutations in KRAS, TP53, SMAD4, and CDKN2A were the most common in both subgroups, among which KRAS and TP53 were the most dominant, with more than 5% in both groups. Missense mutations were the most common, followed by nonsense mutations. By summarizing the somatic mutation situation of cyan module genes, it was found that mutations were mainly SNPs, mainly concentrated in C>T and C>A (Figure 7C). The characteristic risk score was positively correlated with TMB (R = 0.2, p = 0.0096, Figure 7D left), while survival analysis showed that a higher level of TMB was significantly associated with a longer OS (p = 0.02, Figure 7D right).

Subsequently, we compared the copy number variations (CNVs) or mutations of five pain-related MDGs with various immune cells to observe the impact of gene changes in pain-related MDGs on immune infiltration in PC. Here, B cells, CD8^+^ T cells, CD4^+^ T cells, macrophages, neutrophils, and dendritic cells (DCs) were selected to analyze the correlation between CNVs or mutations of genes and these immune cells. Among them, the CNVs of PSMB8, COL17A1, and BICC1 showed no obvious correlation with these six types of immune cells. The CNV of CTRC was significantly positively correlated with macrophages, and the TRIP13 was significantly positively correlated with neutrophils (Supplementary Figure 2A), indicating that the CNV changes of these five key pain-related MDGs had a relatively small impact on immune infiltration in PC. Combining the mutation changes of five pain-related MDGs, it can be seen that PSMB8 was significantly positively correlated with B cells, CD8^+^ T cells, and neutrophils; COL17A1 was significantly positively correlated with B cells, CD8^+^ T cells, and CD4^+^ T cells, and significantly negatively correlated with neutrophils and DCs; no significant correlation was found between the mutations of BICC1 and CTRC and immune cells; the mutation of TRIP13 was significantly positively correlated with CD8^+^ T cells and CD4^+^ T cells, and significantly negatively correlated with neutrophils and DCs (Supplementary Figure 2B). By comprehensively analyzing five MDGs and various immune cells, it was found that low CNVs of these five key MDGs were closely associated with CD4^+^ T cells, B cells, natural killer (NK) cells, etc., while high CNVs were significantly correlated with CD8-naive, natural regulatory T cells (nTreg), neutrophils, etc (Supplementary Figure 2C). Further analysis of the correlation between Single Nucleotide Variant (SNV) of five MDGs and immune infiltration showed that there was no significant correlation between them (Supplementary Figure 2D). At the same time, we also investigated the correlation between the mRNA expression levels of these genes and immune cells (Supplementary Figure 3). Combined with the above research findings, it can be concluded that the mutation is the most significant aspect in their DNA variations in terms of the impact on immune infiltration among these five key MDGs.

In order to observe the distribution of five pain-related MDGs in PC tissues or the immune microenvironment, we utilized the CDCP database and selected five public single-cell analysis data sets, namely, GSM6567161, GSM6567159, GSM663326, GSM728974 and GSM663329, to observe the gene distribution (Figure 7E). Among them, GSM6567161 and GSM6567159 mainly focus on the immune landscape of PC; GSM663326 and GSM663329 are concerned with the comparison between PC and corresponding normal tissues; GSM728974 focuses on tumor microenvironmental changes. It can be seen that the expressions of these genes mainly exist in PP cells, epithelial cells, mesenchymal cells, and acinar cells, which are several types of pancreatic tissue cells.

### 3.7 Analysis of immune function and drug sensitivity of differentially expressed genes

**FIGURE 8:**
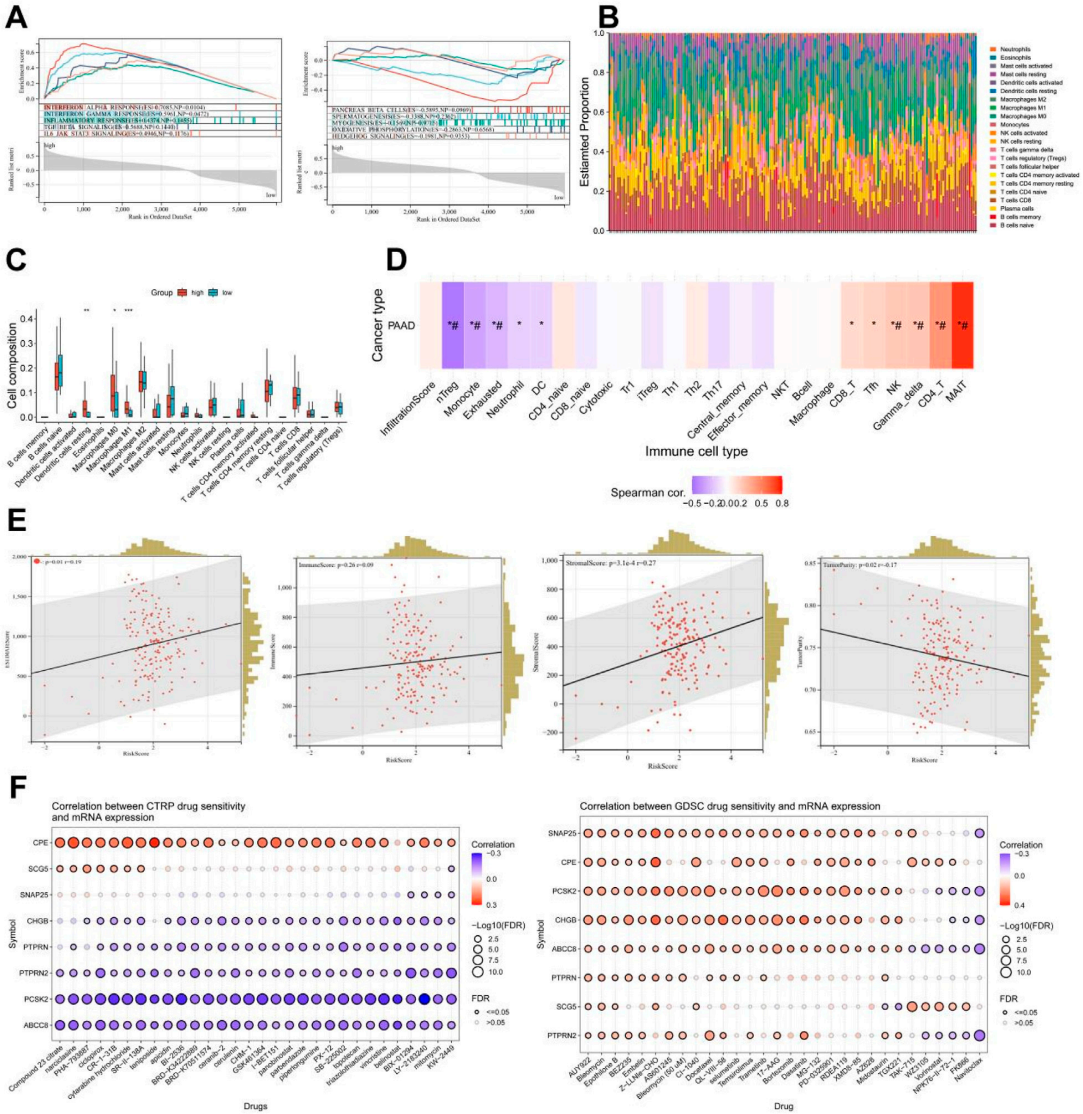
Analysis of immune function and drug sensitivity of differentially expressed genes. **(A)** Enrichment analysis of GSEA-hallmark for risk score model differentially expressed genes. **(B)** Stacked bar plot of estimate scores for 22 kinds of immune cells of differentially expressed genes. **(C)** Boxplot of scores for 22 kinds of immune cells of differentially expressed genes. **(D)** Immune cell infiltration and GSVA enrichment analysis scores of eight key genes (*: p-value < 0.05; #: FDR < 0.05). **(E)** Correlation analysis between risk score and stromal cell score (StromalScore), immune cell score (ImmuneScore), total score (ESTIMATEScore, the sum of stromal cell score and immune cell score), tumor purity (TumorPurity) of differentially expressed genes. **(F)** Drug sensitivity analysis results from CTRP and GDSC websites.

Based on the aforementioned research, it can be discovered that the pain risk subtypes constructed by the MDGs exhibit significant correlations with cellular immune functions. Through the GSEA-hallmark enrichment analysis of 5,962 differentially expressed genes in the risk scoring model, it was shown that the high-risk subgroup was associated with interferon-α response, interferon-β response, immune response, TGF-BETA signaling and IL6-JAK-STAT3 signaling, while the low-risk subgroup was associated with pancreas beta cell, spermatogenesis, myogenesis, oxidative phosphorylation and hedgehog signaling (Figure 8A). The analysis results of 22 types of immune cells for differentially expressed genes indicated that there were significant differences in dendritic cells resting, Macrophages M and Macrophages M1 cells (Figures 8B,C). After performing immune cell infiltration and GSVA enrichment scoring on the previously screened MCODE differentially expressed genes, it was found that they were closely related to multiple immune cells (Figure 8D). By analyzing the correlations between PC ESTIMATEScore, ImmuneScore, StromalScore and TumorPurity and the risk score, it can be seen that the high-risk score was significantly positively correlated with ESTIMATEScore and StromalScore, but negatively correlated with TumorPurity (Figure 8E). The analysis of MCODE gene immune cells showed a positive correlation between gene expression and immune cell function (Supplementary Figure 4). Due to the downregulation of gene expression in high-risk subtypes, it suggests a decrease in cellular immune function and an increase in the proportion of immune infiltration. This indicates that in the high-risk subgroup of PC, the proportions of immune cells and stromal components are higher, suggesting a higher likelihood of immune infiltration. Combined with the drug sensitivity analysis results from CTRP and GDSC websites, the drug sensitivity predictions of different tumor chemotherapy drugs can be seen (Figure 8F).

### 3.8 Single-cell analysis of key MCODE genes in the pancreas or nerves

**FIGURE 9:**
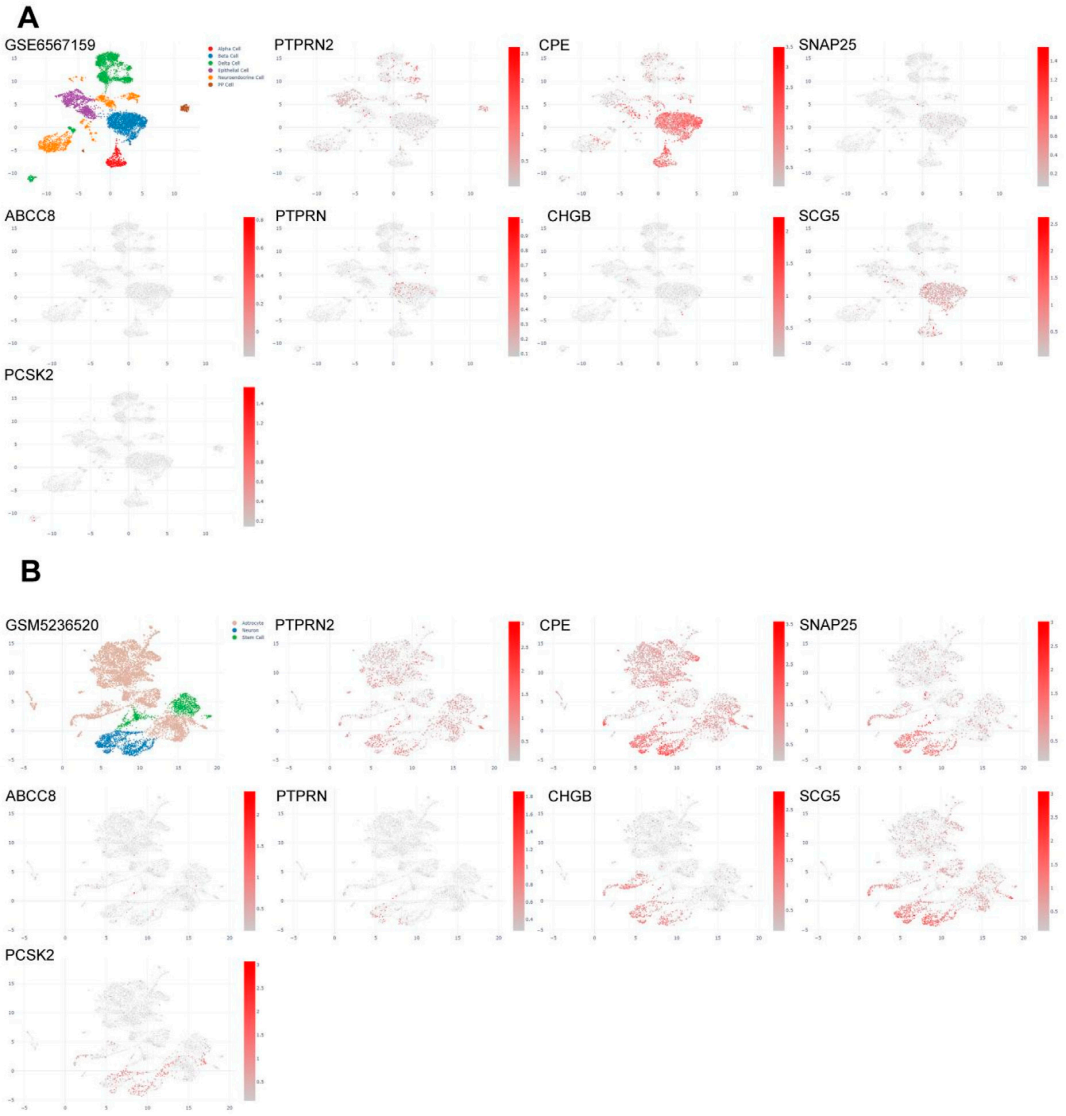
Single-cell analysis of eight key differentially expressed genes in pancreas or nerves. **(A)** Gene expression profiles of single-cell analysis of eight key differentially expressed genes in the pancreas (GSM6567159), which are, in sequence, PTPRN2, CPE, SNAP25, ABCC8, PTPRN, CHGB, SCG5, PCSK2. **(B)** Gene expression profiles of single-cell analysis of eight key differentially expressed genes in nerves (GSM5236520), which are, in sequence, PTPRN2, CPE, SNAP25, ABCC8, PTPRN, CHGB, SCG5, PCSK2.

In the development mechanism of PC pain, besides inflammatory pain caused by changes in immune function, neuropathic pain caused by tumor tissue compressing nerves is also crucial. Combined with previous studies, it can be seen that the risk scoring model constructed by pain-related MDGs may have certain correlations with early metastasis of pancreatic tumors. The selected differentially expressed genes play important roles in synapses related to nerve signal transmission and voltage-gated ion channels. Therefore, we further analyzed their expression distributions in the pancreas and nerves (Figure 9). GSM6567159 is a single-cell analysis data set of the immune landscape of PC, and GSM523652 is a single-cell transcription atlas of the human spinal cord. It can be seen that MCODE is expressed in different degrees not only in pancreatic tissues but also more in nerve cells, providing further evidence for the influence of perineural infiltration of PC on pain.

## 4 Discussion

**FIGURE 10:**
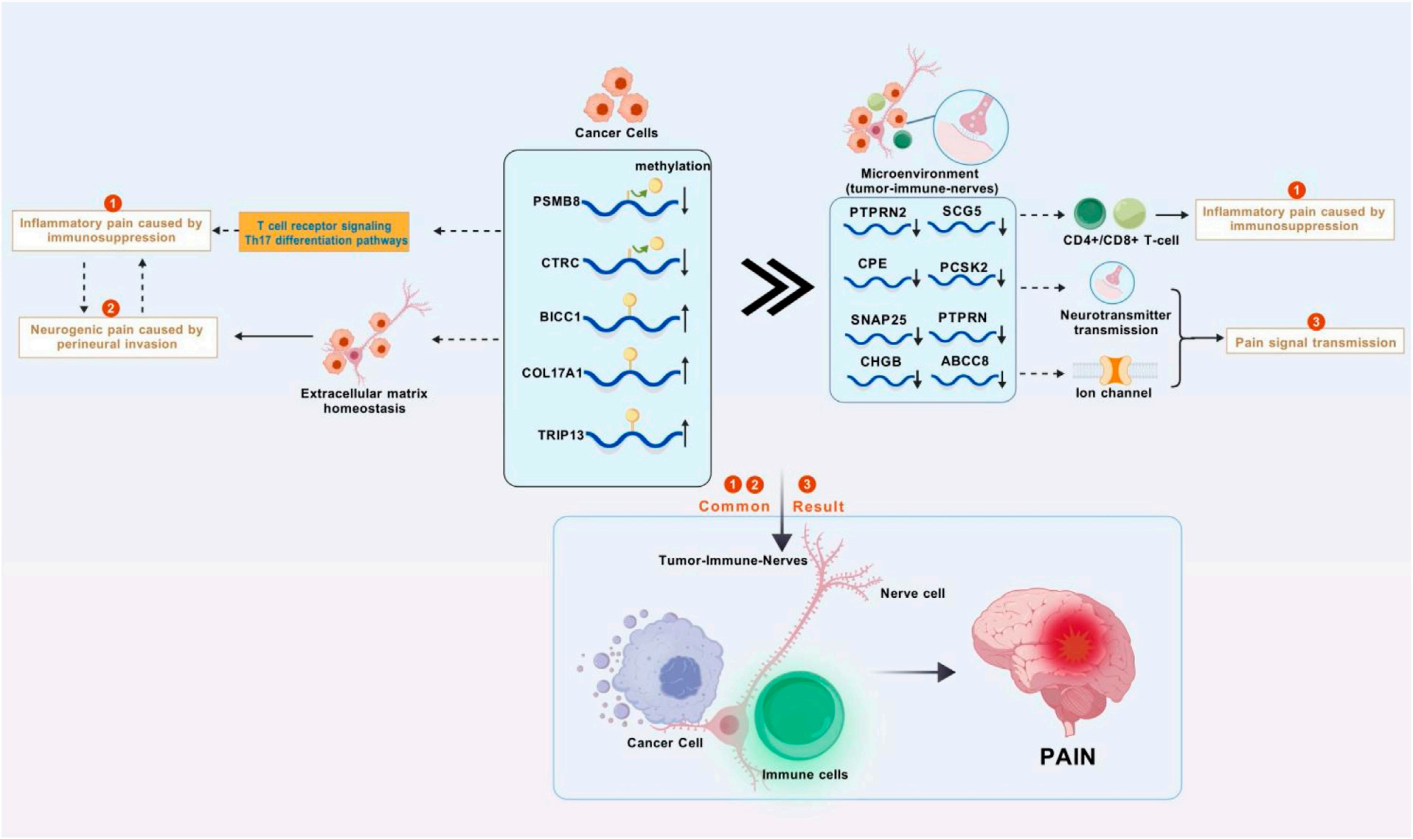
Research mechanism diagram. Abnormal methylation genes in pancreatic cancer cells disrupts the immune system and the homeostasis of the extracellular matrix, leading to inflammatory tumors compressing nerves and causing inflammatory pain and neuropathic pain. These abnormal methylation genes also affect the tumor-immune-neural microenvironment through synaptic signaling, resulting in further enhancement of inflammation, continuous transmission of pain signals, and ultimately persistent and severe cancer pain (Figure was created with BioGDP.com).

In recent years, the global incidence of PC has shown a sharp increase. As one of the leading causes of cancer deaths, the diagnosis and treatment of PC are particularly important (Li et al., 2025). Pain is a significant factor affecting the quality of life for PC patients and is crucial for patient outcomes (Tarasiuk et al., 2023). The main mechanisms of PC pain include direct compression and infiltration of surrounding nerves by the tumor, as well as the release of inflammatory mediators (Zhu et al., 2024). Previous studies have indicated that cancer pain primarily involves genetic and epigenetic regulation. Epigenetic regulation, including DNA methylation, histone modifications, and non-coding RNAs, affects neuroinflammation, neuronal sensitization, and pain transmission by regulating the expression of pain-related genes or the activity of signaling pathways (Ni et al., 2024). Based on differences in the degree of PNI and inflammatory mediators/chemokines between PC and adjacent normal tissues, we extracted relevant data from TCGA for PC and adjacent normal tissues. Using the MethylMix software package, 471 MDGs were identified, and five pain-related MDGs (PSMB8/COL17A1/BICC1/CTRC/TRIP13) significantly associated with overall survival (OS) were finally selected for prognostic stratification of PC patients (HR = 3.83, p < 0.001). High-risk patients exhibit pronounced immunosuppression, with enriched functions in synaptic vesicle cycling and signal transmission. Combined with single-cell analysis, it was observed that differentially expressed genes are widely distributed in both the pancreas and nerves, suggesting that pain-related MDGs not only affect the immune microenvironment but also regulate signal transmission between tumors and nerves. This is a key process in initiating visceral nociception. Our study finds that the complex pathogenesis of PC pain extends beyond mechanical compression and inflammatory mediator release, involving intricate immuno-neural genetic crosstalk (Figure 10).

Epigenetic modifications can affect gene expression at multiple levels, including transcription, splicing, stability, and translation, thereby influencing the physiological and pathological functions of cells. These modifications primarily consist of DNA methylation, histone modifications, non-coding RNAs, RNA modifications, and chromatin remodeling (Zheng et al., 2025). Among these, DNA methylation is the earliest and most extensively studied epigenetic modification. Research has found that DNA methylation can inhibit or activate genes related to pain perception, conduction, and regulation, thereby altering nerve cells’ response to pain (Sun et al., 2019). In a model of spinal nerve ligation (SNL) in male Sprague Dawley rats, a persistently low level of hypomethylation was observed at CpG sites in the DRG. Reducing DNA methylation can cause pain hypersensitivity, while increasing DNA methylation can alleviate neuropathic pain (Garriga et al., 2018). DNA methylation can also regulates neural plasticity, affecting connections and communication between nerve cells (Pratt et al., 2022). In oral cancer, abnormal demethylation of various tumor suppressor genes and hypermethylation of nerve growth factors have been found to be involved in the development of PNI (Hurnik et al., 2022). In this study, MDGs were identified using the MethylMix package combined with gene expression profiling and methylation data analysis. These genes showed a negative correlation between methylation and mRNA expression, and differences in DNA methylation levels were observed between cancer and normal samples, resulting in 471 MDGs. Further screening yielded 26 MDGs related to PC pain, followed by GO and KEGG enrichment analyses. GO enrichment analysis revealed three main functions of these epigenetic regulators: (1) structural regulation through extracellular matrix organization, (2) transcriptional reprogramming via DNA-binding activator activity, and (3) neuronal excitability modulation through ligand-gated cation channels. KEGG pathway dissection uncovered the convergence of the immune-pain axis, with significant enrichment in T cell receptor signaling and Th17 differentiation pathways. Notably, our findings maybe suggest a novel mechanistic link: (1) epigenetic silencing of ion channels may disrupt pain signal transduction, (2) methylation-mediated immune modulation (Th17 pathway genes) indicates tumor microenvironment crosstalk, and (3) Methylation of structural genes (COL family) may alter the PNI ability of tumors. The multi-scale integration of methylation drivers with pain biology demonstrates epigenetic coordination between cancer progression and nociceptive signaling for the first time. Further analysis of the 26 genes led to the construction of a risk scoring model, identifying five key genes: PSMB8, COL17A1, BICC1, CTRC, and TRIP13. Among them, PSMB8, an immunoproteasome component, plays a crucial role in inflammation regulation (Yang et al., 2009; Kitamura et al., 2011). Abnormal methylation of PSMB8 has been reported in ovarian, breast, and colorectal cancers (Liew et al., 2018; Siebenkas et al., 2017; Tian et al., 2022). COL17A1, a member of the collagen family, exhibits abnormal promoter methylation leading to overexpression in cervical and epithelial cancers, enhancing tumor invasiveness (Thangavelu et al., 2016). It has also been identified as a potential biomarker for PC prognosis (Huang et al., 2022). BICC1, an RNA-binding protein, influences tumor progression in colon cancer through pathways such as extracellular matrix (ECM) receptor interaction and focal adhesion (Lv et al., 2020). CTRC (chymotrypsin C) encodes a protein crucial for regulating the activation and degradation of trypsinogen and procarboxypeptidase, protecting the pancreas from pancreatitis and influencing pain progression in chronic pancreatitis (Demcsak et al., 2024; Dunbar et al., 2025). TRIP13 encodes a thyroid hormone receptor-interacting protein, also known as a hormone-dependent transcription factor, promoting the progression of pancreatic ductal adenocarcinoma by facilitating tumor tissue growth and metastasis (Afaq et al., 2024). Based on the speculation of regulatory mechanisms combining five genetic features and above, it can be observed that a crucial genetic driver coordinates the regulation among three parties. PSMB8 affects neuroinflammation by regulating the activity of immunoproteasome, while COL17A1 and BICC1 promote the progression of PNI in tumor cells by regulating matrix homeostasis and ECM. The disruption of CTRC destabilizes pancreatic protease, exacerbating substantive nociception, and TRIP13 may influence related pain progression through hormonal regulation. The above research results indirectly suggest that epigenetic dysregulation in PC may be involved in the regulation of cancer pain by affecting immune reprogramming and the nervous system. However, further exploration is needed to determine whether there is a direct connection between epigenetics, immunology, and neurology. The specific internal relationships and molecular mechanisms also require more clinical and functional validation.

In the context of cancer pain, previous studies have predominantly focused on the regulatory mechanisms of pain within the nerve conduction pathway, overlooking the role of the tumor itself and the complex tumor-nerve crosstalk (Xu et al., 2024a). PC tumors consist of malignant tumor cells, stromal cells, immune cells, and other extracellular matrix components. Past research suggests that dynamic remodeling of the tumor microenvironment is critical to the progression of cancer pain. During malignant proliferation, mechanical compression of surrounding tissues by the tumor mass causes injury, and the release of various inflammatory factors (such as prostaglandins, interleukins, and tumor necrosis factors) can damage nerve endings, triggering inflammatory pain (Capodanno and Hirth, 2023). Metabolites of cancer cells, such as lactic acid, induce local acidosis and activate ASICs/TRPV1 ion channels (Qian et al., 2021). Additionally, tumor cells can directly invade nerves, causing structural damage (Gil et al., 2010). More importantly, studies have confirmed that tumor-associated macrophages (TAMs) can activate Schwann cells through the bFGF/PI3K/Akt/c-myc/GFP pathway. Schwann cells secrete IL-33, recruiting macrophages into the perineural environment and promoting their M2 tumor-promoting polarization (Zhang et al., 2024). The positive feedback loop between the two has a significant impact on the PNI process in PC, indicating crosstalk between the immune system and nervous system in the development of cancer pain. Differential expression analysis of 22 immune cell types in this study showed that pain risk subtypes are associated with dendritic cell dysfunction and M0/M1 macrophage imbalance. Immune cell infiltration and GSVA enrichment scores indicated that MCODE differentially expressed genes are negatively correlated with various immunosuppressive cells such as nTregs, neutrophils, and DC cells, and positively correlated with immune effector cells such as CD8 T, Tfh, NK, CD4 T, and MAIT. Previous research and analysis results suggest that the tumor parenchymal microenvironment, rather than secondary neural effects, regulates PC pain progression through neuro-immune interactions. However, the epigenetic regulatory mechanisms involved still require further exploration.

By conducting a WGCNA on the differential genes of different pain risk subgroups, the cyan module containing 89 differential genes most relevant to the T staging was obtained. The results from KEGG suggest that these differential genes are involved in synapse vesicle recycling, insulin secretion, diabetes, and other pancreas-related functions. GO analysis revealed that the biological processes of these differential genes are primarily enriched in signal release, hormone secretion and transport, and synaptic plasticity regulation. Synaptic plasticity refers to the dynamic changes in synaptic transmission efficiency, including long-term potentiation (LTP) and long-term depression (LTD) (Costenla et al., 2001). In pain pathways (such as the spinal dorsal horn, thalamus, and cortex), LTP can lead to hyperalgesia and allodynia by enhancing synaptic transmission efficiency (Rygh et al., 2002). In chronic pain, glutamic acid triggers calcium influx through NMDA receptors, activating downstream signals (such as CaMKII, PKC), inducing synaptic LTP, and amplifying pain signals (Bliss et al., 2016). The cellular components and molecular functions of the cyan module are mainly enriched in vesicle transport, potassium ion channels, and related transporter activity regulation. Vesicle transport is responsible for the synthesis, storage, and release of neurotransmitters (such as glutamate, substance P, and CGRP) (Tao et al., 2022). In nerve damage or inflammation, dysregulation of vesicle transport-related proteins (such as SNARE complexes, synaptotagmin) leads to excessive release of glutamate and substance P, enhancing postsynaptic neuronal excitability (Pan and Rutecki, 2014). CGRP and substance P participate in neurogenic inflammation through vesicle release, further activating peripheral and central pain pathways (Meseguer et al., 2014). Botulinum toxin (BoNT) inhibits vesicle release by cleaving SNARE proteins and has been used to treat migraine and neuropathic pain (Vacca et al., 2020). Potassium ion channels affect the generation and transmission of pain signals by regulating membrane potential repolarization and neuronal excitability (Loucif et al., 2018). Thus, it can be seen that gene expression differences among different pain subtypes are mainly concentrated in various steps of pain-related signal transmission.It can be seen that the differentially expressed genes of pain risk subtypes affect various nodes of pain signal transmission through dynamic regulation of neurotransmitters and synaptic efficacy reprogramming. Since differentially expressed genes are also involved in insulin secretion and diabetes-related pathways, it suggests to some extent the organ-specific coupling of neuroendocrine-nociceptive transmission. The above studies indicate to some extent the potential connection between epigenetic regulation and tumor-neural crosstalk, and the specific mechanism is also the focus of further exploration.

Using cytoscope to screen MCODE genes from the cyan module, eight most significant pain-related differential genes were identified, namely, PTPRN2, CPE, SNAP25, ABCC8, PTPRN, CHGB, SCG5, and PCSK2. TIMER analysis revealed that the expression levels of these genes in PC are mostly negatively correlated with tumor purity, indicating high expression in the tumor microenvironment. However, PTPRN2 showed a positive correlation with tumor purity, suggesting relatively higher expression in the tumor. Therefore, the GSM6567159 PC immune landscape dataset and the GSM5236520 human spinal cord dataset were selected for single-cell analysis of MCODE genes. Among them, PTPRN2 encodes a protein tyrosine phosphatase receptor expressed in endocrine and neuronal cells (Sorokin et al., 2015), playing a role in exocytosis and affecting synaptic plasticity in neurons (Jiang et al., 1998). The single-cell analysis in this study found that PTPRN2 is distributed in pancreatic delta cells and mesenchymal cells, while in the spinal cord, it is evenly distributed in astrocytes, neurons, and stem cells. Immune cell function analysis suggested a positive correlation between PTPRN2 expression levels and various cells such as B cells, CD8^+^ T cells, and CD4^+^ T cells in PC. In high pain risk subtypes, PTPRN2 expression levels decrease, leading to reduced cellular immune function. CPE encodes an enzyme widely present in neuroendocrine cells, playing a key role in the processing of peptides and hormones. Studies suggest that CPE can promote the entry of eosinophil cationic protein into neuroendocrine cells (Wu et al., 2004). In the pancreas, CPE may be involved in the post-processing of hormones such as insulin and glucagon, closely related to pancreatic function (Chen et al., 2023). Single-cell analysis of the pancreas showed that CPE is abundantly distributed in pancreatic Alpha and Beta cells, as well as in astrocytes, neurons, and stem cells. In high pain risk subtypes, CPE expression levels decrease, positively correlating with the function of various immune cells such as B cells, CD8^+^ T cells, and CD4^+^ T cells, leading to reduced cellular immune function. Studies have found that SNAP25 increases the release of excitatory neurotransmitters (such as glutamic acid) in the dorsal horn of the spinal cord through presynaptic mechanisms, potentially leading to an imbalance of excitatory/inhibitory neurotransmitters, thus mediating the development of neuropathic pain (Tafoya et al., 2006). In this study, SNAP25 is less distributed in PC, mainly in neuronal cells, positively correlating with immune cell function. The ABCC8 gene encodes the SUR1 subunit of the ATP-sensitive K+ channel (KATP) in pancreatic beta cells. Mutations in this channel can lead to congenital hyperinsulinism (CHI), a disease associated with excessive or unregulated insulin secretion. Although it does not directly cause pain, it may have indirect links to the physiological functions of the pancreas and related pain states (Gloyn et al., 2003). Single-cell analysis showed less distribution of ABCC8 in pancreatic tissue and neurons. In high pain risk subtypes, ABCC8 expression levels decrease, positively correlating with the function of various immune cells such as B cells, CD8^+^ T cells, and CD4^+^ T cells, leading to reduced cellular immune function. PTPRN is a receptor-type tyrosine phosphatase primarily involved in regulating secretory granule control in neuroendocrine cells and islet beta cells, affecting the release of hormones (such as insulin) and neurotransmitters (Harash et al., 2012) Chromogranin B (CHGB) is a marker protein of secretory granules, involved in regulating the storage, processing, and release of neuropeptides and catecholamines (Fung et al., 2008) SCG5 is a key component of secretory granules, involved in regulating the processing and secretion of neuropeptides and hormonesPC (Avgan et al., 2023) SK2 is responsible for cleaving inactive precursor proteins (such as neuropeptide precursors) into active forms (such as endorphin, proinsulin)ese (Podvin et al., 2018) four gene-encoded proteins all play a role in the production and transmission of neurotransmitters. Single-cell analysis revealed that PTPRN and SCG5 are distributed in pancreatic beta cells, while the expression of CHGB and PCSK2 is relatively low. In spinal cord tissue, PTPRN expression is low, while CHGB, SCG5, and PCSK2 are all enriched in neuronal cells. TIMER immune infiltration analysis showed that these genes regulate cellular immune function through the tumor microenvironment. Thus, it can be inferred that in the risk subgroups of pain-related MDGs in PC, differential gene expression primarily regulates neurotransmitter processing and transmission steps in neural signals through the tumor microenvironment, further confirming the mutual crosstalk between tumor and nerves.Immune function and single-cell resolution reveal that PC pain-related MCODE genes constitute neurotransmitter processing hubs that mediate immune-neural interactions through the tumor microenvironment. Among them, PTPRN2/CPE/SCG5/PCSK2 regulate immune function based on the neuroendocrine system, SNAP25/CHGB/PTPRN affect neural signaling based on synaptic signal transmission, and ABCC8 indirectly participates in pain regulation based on the physiological characteristics of ion channels. This study proposes for the first time at the single-cell level that tumor epigenetics hijack both neuroendocrine and immune systems by affecting the microenvironment, leading to the development and progression of cancer pain. It focuses on pancreatic parenchymal damage rather than nerve transmission as the key to PC pain, providing a new perspective for the mechanistic study of cancer pain.

The current clinical research progress has indirectly validated the “DNA methylation-immunity-neuro” interaction model speculated in this study to some extent. This is specifically manifested in the combined therapy of DNA methyltransferase inhibitors (DNMTi) and immune checkpoint inhibitors (ICI), as well as analgesic research on drugs targeting MCODE genes. Studies have found that DNMTi can reverse tumor immunosuppression by reactivating antitumor immune signals or reshaping the expression of immune checkpoints. In ovarian cancer, Peng et al. discovered that DNMTi enhances the therapeutic effect of anti-PD-L1 by reactivating Th1-type chemokines and increasing T-cell infiltration (Peng et al., 2015). La ure Ricard et al. treated nine patients with relapsed/refractory Angioimmunoblastic T-cell lymphoma (AITL) using a combination of 5-azacytidine (one of the DNMTi) and nivolumab (anti-PD-1 checkpoint blockers). The overall response rate reached 78% with reduced chemotherapy toxicity (Ricard et al., 2024). In breast cancer, combined therapy with PD-1 antibodies and guadecitabine (one of the DNMTi) can enhance MHC-I expression and increase CD8 T-cell infiltration in TME, thereby enhancing the therapeutic effect of PD-1 antibodies (Luo et al., 2018). In mouse models of melanoma, colorectal, breast, and ovarian tumors, the combined treatment of DNMTis and anti-CTLA-4 ICI can extend survival to some extent (Guo et al., 2023). Cl inical drug research on MCODE genes has found that potassium channel openers such as retigabin and flupirtine are effective in treating neuropathic pain caused by nerve ligation models, inflammatory pain caused by formaldehyde, and visceral pain caused by capsaicin (Busserolles et al., 2016). Perineural injection of botulinum toxin type A (pBONT-A) targeting SNAP25 under ultrasound guidance can alleviate pain associated with PC nerve infiltration (Meyer-Frießem et al., 2019).

Through bioinformatics analysis, this study proposes an innovative regulatory framework: There is a significant correlation between DNA methylation changes in PC pain, the progression of PNI, and the remodeling of the immune microenvironment. At the mechanistic level, it has been found that methylated driver genes are associated with pathological processes such as neural plasticity and immunosuppression, suggesting that epigenetic reprogramming may participate in the pathogenesis of pain through PNI-related nerve damage and immune remodeling. However, the causal relationship between the two still needs experimental verification. The constructed “epigenetic-immune-neural” three-dimensional network provides a testable hypothesis for subsequent mechanistic studies. However, there are several limitations to this study: Firstly, studies based on public datasets have insufficient sample diversity and lack multi-center validation. Secondly, the lack of *in vivo* models prevents the analysis of the spatiotemporal dynamics of pain signals. Finally, the discovery of correlation has not been verified through functional experiments, and the specific molecular mechanism cannot be clarified. Future research should focus on establishing an *in situ* PC model for neural epigenome editing, tracking the dynamic evolution of methylation during the PNI process, and determining the proportional weight of immune and neural contributions to the pain phenotype.

## Data Availability

Publicly available datasets were analyzed in this study. The data presented in the study are deposited in the TCGA repository, MSigDB and GEO database, the accession number are respectively: https://portal.gdc.cancer.gov/projects/TCGA-PAAD, HP_PAIN (M38128), GSE183795.
