# Supplementary material for: Pain - related methylation driver genes affect the prognosis of pancreatic cancer patients by altering immune function and perineural infiltration
Source: Front Genet. 2025 Oct 8;16:1600883. doi: 10.3389/fgene.2025.1600883 (PMC12540144; doi:10.3389/fgene.2025.1600883)
Supplement: Supplementary file 3 [file DataSheet3.docx]

Supplementary figure 1


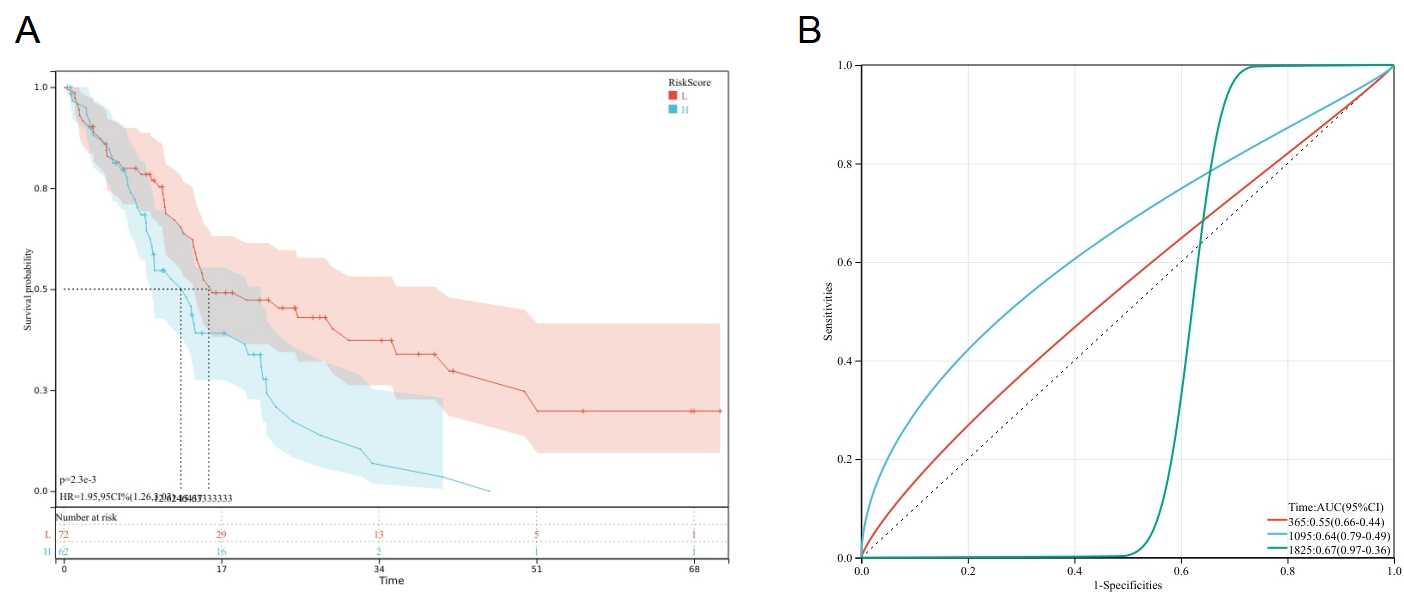


Supplementary figure 1 KM curve and ROC curve of GSE183795 dataset

(A) KM curve of GSE183795 dataset.(B) ROC curve of GSE183795 datase

Supplementary figure 2
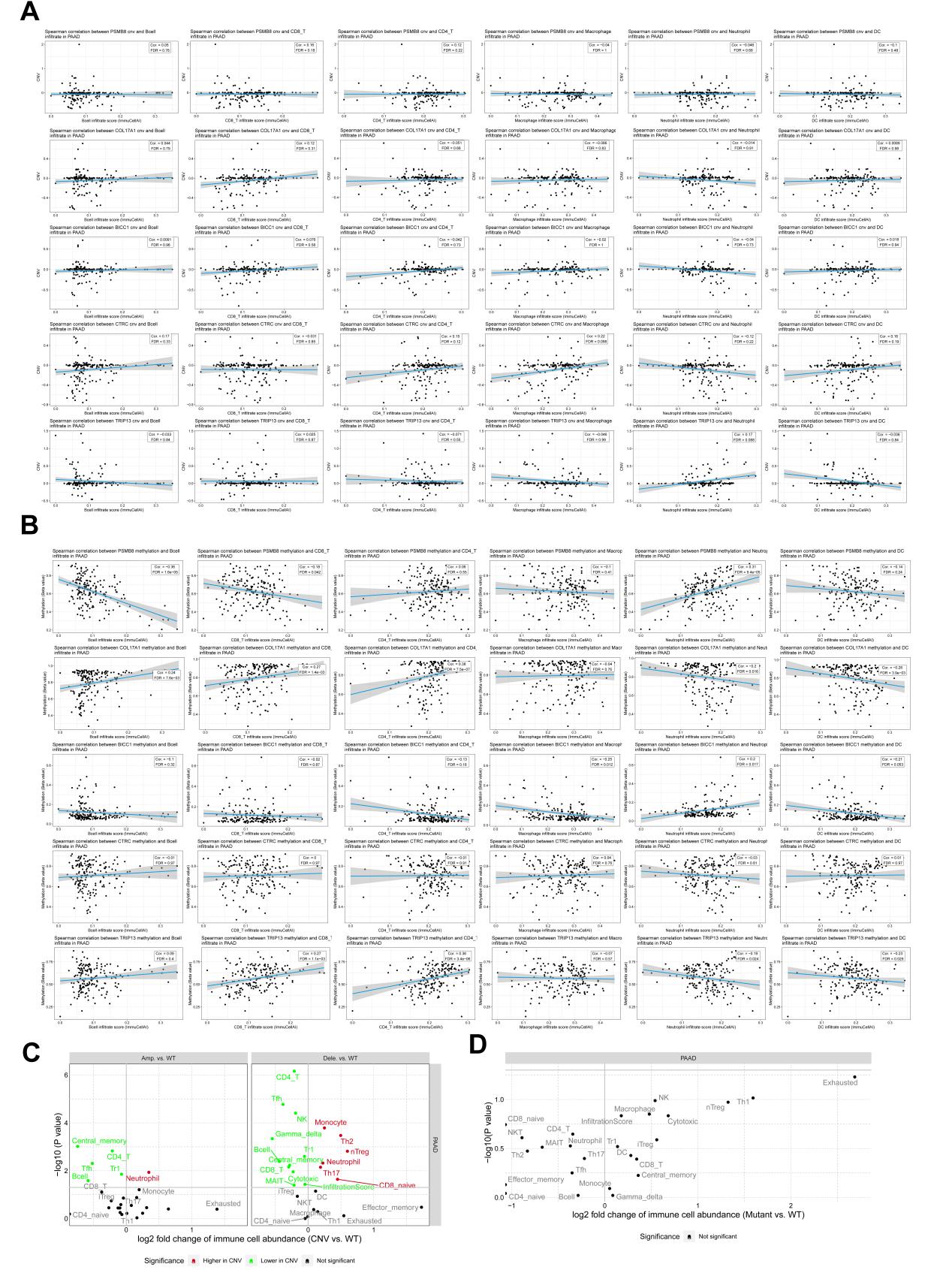


Supplementary figure 2 Correlation between CNV, methylation, and immune infiltration of key pain related methylation driver genes

1. Correlation analysis between CNV and immune infiltration in key pain related MDGs.(B) Correlation analysis between methylation and immune infiltration in key pain related MDGs. (C) Comprehensive analysis of CNV and immune infiltration in key pain related MDGs.(D) Comprehensive analysis of SNV and immune infiltration in key pain related MDGs.

Supplementary figure 3


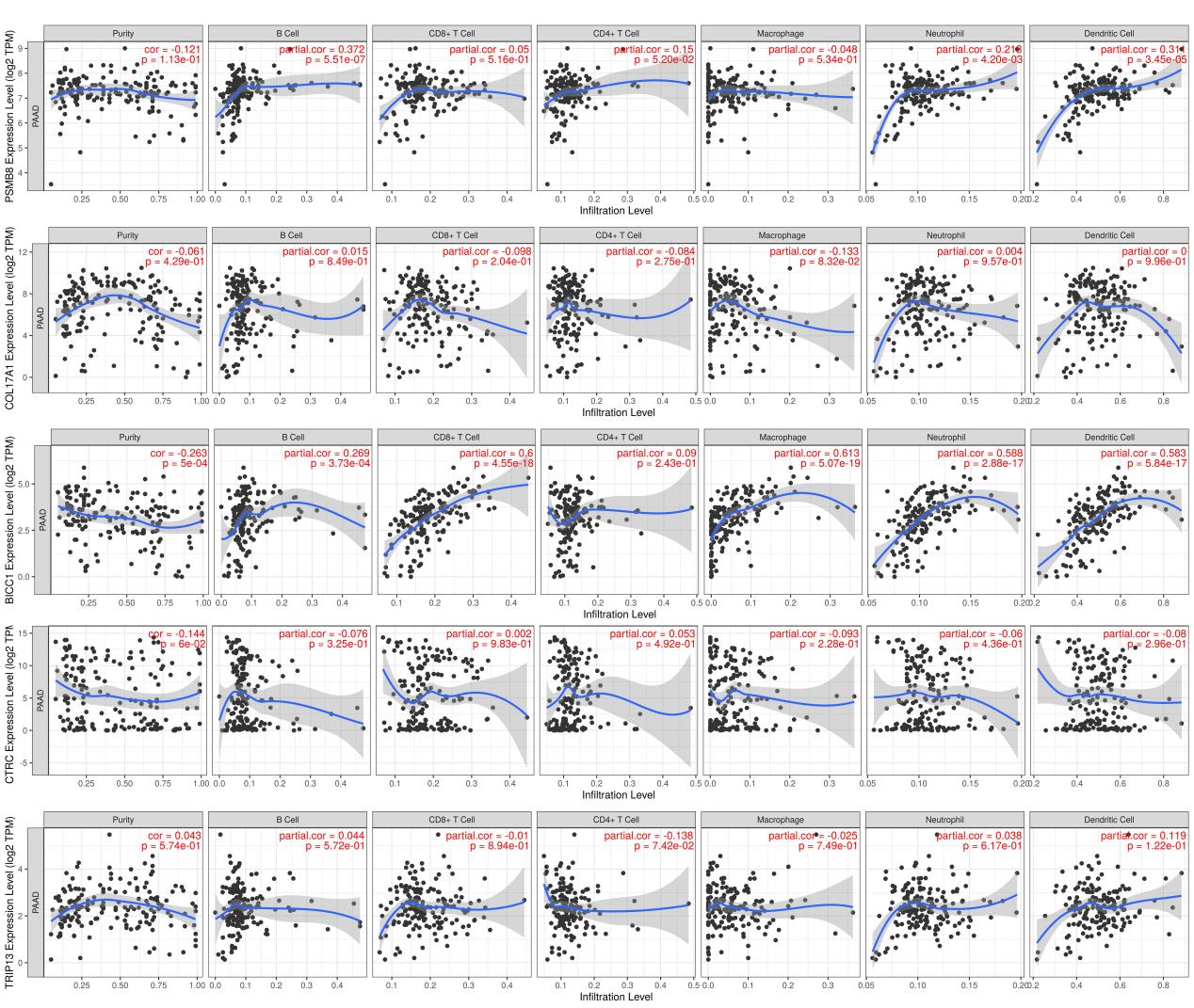


Supplementary figure 3 Correlation Analysis between mRNA Expression of Key Pain Related MDGs and Immune Infiltration.

Supplementary figure 4


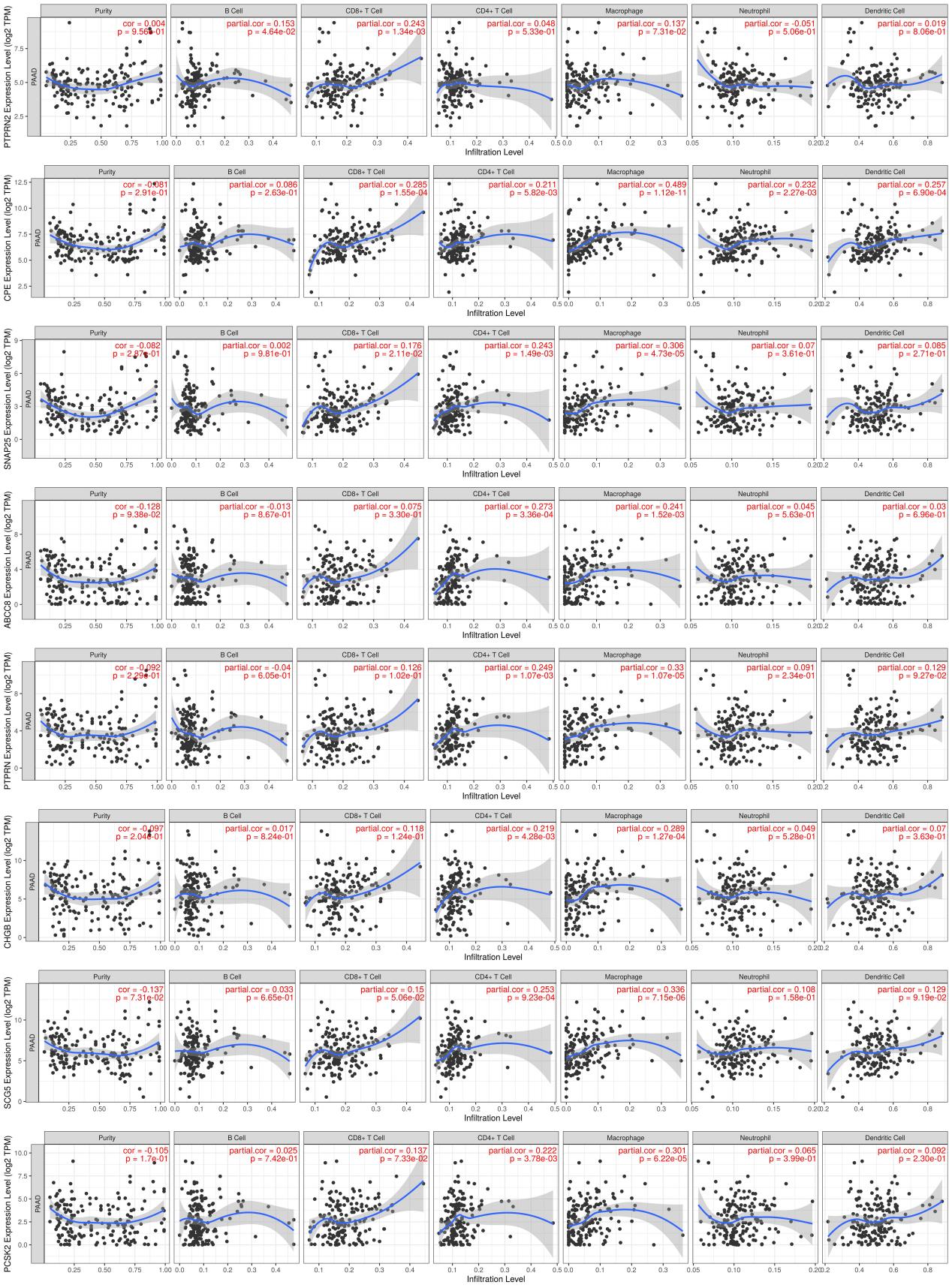


Supplementary figure 4 Correlation Analysis between mRNA Expression of MCODE Differential Genes and Immune Infiltration
